# Evaluating Immune-Inflammatory Indices for Risk Stratification in Cardiovascular Disease: An Umbrella Review of Systematic Reviews and Meta-Analyses

**DOI:** 10.3390/diagnostics15222862

**Published:** 2025-11-12

**Authors:** Hanxin Liu, Pingwu Wang, Lik Hang Wu, Fan Wu, Xinya Zhou, Yuhan Li, Hui Su, Jiayi Zang, Xinchen Ji, Xueling Xiao, Ya-Ke Wu, Leroy Sivappiragasam Pakkiri, Chester Lee Drum

**Affiliations:** 1 Yong Loo Lin School of Medicine, National University of Singapore (NUS), NUHS Tower Block, 1E Kent Ridge Road Level 11, Singapore 119228, Singapore; hanxin_liu@u.nus.edu (H.L.); likhang_wu@u.nus.edu (L.H.W.); jiayi_zang@u.nus.edu (J.Z.); ji.xinchen@u.nus.edu (X.J.); mdclspk@nus.edu.sg (L.S.P.); mdccld@nus.edu.sg (C.L.D.); 2 Cardiovascular Research Institute (CVRI), National University Health System (NUHS), 14 Medical Drive, MD6 Level 8, Singapore 117599, Singapore; 3 Department of Medicine, Yong Loo Lin School of Medicine, National University of Singapore (NUS), Singapore 119228, Singapore; 4 Xiangya School of Nursing, Central South University, 172 Tongzipo Road, Changsha 410013, China; pingwu_wang@126.com; 5 College of Medicine and Health Science, Wuhan Polytechnic University, 68 Xuefu South Road, Changqing Garden, Wuhan 430023, China; wufan_1006@126.com (F.W.); zhouxinya611@163.com (X.Z.); huisu_sofia1003@163.com (H.S.); 6 Department of Operating Room, Union Hospital, Tongji Medical College, Huazhong University of Science and Technology, Wuhan 430022, China; yuhan_li0726@163.com; 7 School of Nursing, University of North Carolina at Chapel Hill, Chapel Hill, NC 27599, USA; 8 Department of Psychiatry, University of North Carolina at Chapel Hill, Chapel Hill, NC 27599, USA; 9 Department of Biochemistry, National University of Singapore (NUS), 8 Medical Drive, MD7, Singapore 117596, Singapore

**Keywords:** immune-inflammatory indices, cardiovascular diseases, prognostic biomarkers, umbrella review

## Abstract

**Background/Objectives:** Although systematic reviews and meta-analyses have examined immune-inflammatory indices in cardiovascular disease (CVD), the evidence remains scattered and inconsistent. This umbrella review aims to synthesize findings and evaluate the overall predictive value of these indices for clinical outcomes. **Methods:** We systematically searched PubMed, Cochrane Library, Web of Science, Embase, Scopus, and Medline for systematic reviews with meta-analyses assessing neutrophil-to-lymphocyte ratio (NLR), systemic immune-inflammation index (SII), platelet-to-lymphocyte ratio (PLR), and systemic inflammation response index (SIRI) in patients with CVD. Study quality and certainty of evidence were appraised using AMSTAR-2 and GRADE, respectively. **Results:** A total of 35 meta-analyses covering 106 unique outcomes were included, of which 87 showed significant associations. Elevated NLR and SII were consistently linked to higher risks of CVD mortality, major adverse cardiovascular events, myocardial infarction, heart failure, and stroke. PLR and SIRI were primarily associated with poor recovery from stroke and increased mortality in ST-elevation myocardial infarction. Specifically, the methodological quality of the included reviews was generally moderate to high according to AMSTAR-2, whereas none of the associations reached high certainty based on GRADE, with most rated as low or very low and about one-quarter as moderate certainty. **Conclusions:** The overall certainty of evidence remains limited according to GRADE, alongside methodological heterogeneity, population variability, and inconsistent thresholds that further restrict the direct applicability of these findings in clinical practice. Nevertheless, available evidence indicates that elevated immune-inflammatory indices are likely associated with worse clinical outcomes in patients with CVD. Future research should prioritize establishing standardized cutoffs, improving methodological consistency, and validating these indices across diverse populations to support their integration into clinical risk-stratification frameworks.

## 1. Introduction

Cardiovascular disease (CVD), including coronary artery disease (CAD), peripheral arterial disease (PAD), heart failure (HF), and stroke, remains a leading cause of death and disability worldwide [1,2,3]. In 2021, CVD accounted for 20.5 million deaths, comprising nearly one-third of all global fatalities [4]. Moreover, CVD is also a major contributor to disability-adjusted life years (DALYs), reflecting both premature mortality and years lived with disability. In 2019, ischemic heart disease and stroke remained the leading causes of DALY’s globally, accounting for 7.2% and 5.7% of total DALYs, respectively [2]. The burden is particularly severe in low- and middle-income countries, where over 75% of CVD-related deaths occur, exacerbated by limited healthcare access and disparities in preventive strategies. In Southeast Asia, CVD contributes to approximately one-quarter of all non-communicable disease (NCD)-related deaths, according to the World Health Organization (WHO) [5]. Notably, a significant proportion of these deaths are considered preventable [6].

Prognostic assessment in CVD is essential for identifying high-risk individuals, guiding therapeutic decisions, and improving long-term clinical outcomes. However, despite advances in evidence-based therapeutic strategies aimed at reducing CVD-related morbidity and mortality, patients remain at significant risk of recurrent cardiovascular events [7,8]. The one-year recurrence rate of major adverse cardiovascular events (MACE) in post-myocardial infarction (MI) patients exceeds 20%, while the five-year mortality rate for HF patients remains over 50%, underscoring the urgent need for improved risk stratification tools [9]. Traditional prognostic models, which primarily rely on clinical risk factors, comorbidities, and imaging-based assessments, often fail to account for the inflammatory and immune mechanisms that drive disease progression [10]. Given these limitations, more comprehensive prognostic tools are needed to enhance risk stratification and clinical management, ultimately improving patient outcomes in individuals with established CVD.

Inflammation and immune dysregulation play a pivotal role in CVD pathogenesis, contributing to vascular dysfunction, atherosclerosis, and thrombosis [11,12]. Key immune cells including lymphocytes, neutrophils, platelets, and monocytes are actively involved in these processes. Neutrophils, as central mediators of innate immunity, drive inflammation through the release of reactive oxygen species and pro-inflammatory cytokines, which promote endothelial dysfunction and atherosclerotic plaque formation [13,14]. In contrast, lymphocytes are crucial for immune surveillance and regulation, with lymphopenia often signaling immune impairment and heightened systemic inflammation, both hallmarks of CVD progression. Platelets, traditionally recognized for their role in hemostasis, actively engage in inflammatory processes by interacting with leukocytes and endothelial cells, thereby facilitating thrombosis and worsening vascular injury [15]. Similarly, monocytes contribute to CVD pathophysiology by differentiating into macrophages and foam cells within atherosclerotic plaques, perpetuating chronic inflammation and promoting plaque instability [16,17]. Reflecting this cellular imbalance, several composite blood-based indices have been developed, including the neutrophil-to-lymphocyte ratio (NLR), systemic immune-inflammation index (SII), platelet-to-lymphocyte ratio (PLR), and systemic inflammation response index (SIRI). These indices integrate multiple components of immune and inflammatory activity, capturing the complex interplay between systemic inflammation and immune regulation in CVD [18]. These indices have gained recognition for their potential in cardiovascular risk assessment, demonstrating value in early risk stratification, recurrence prediction, treatment efficacy evaluation, and personalized therapy [19,20,21,22,23,24,25]. Given their accessibility through routine blood tests, these biomarkers offer a cost-effective, repeatable, and non-invasive approach to evaluating CVD prognosis and treatment response. Compared to traditional risk models that primarily focus on cholesterol levels, blood pressure, and diabetes status, inflammatory indices provide a dynamic assessment of disease activity, reflecting the interplay between the immune system and cardiovascular function. Though numerous systematic reviews and meta-analyses were performed on the relationship between immune-inflammatory index and clinical outcomes of patients with CVD, findings were inconsistent [26,27,28,29]. Current research tends to explore the prognostic value of individual inflammatory markers, such as C-reactive protein (CRP) or interleukin-6, in predicting cardiovascular events, often without comprehensive comparison [30,31]. However, immune-inflammatory indices differ from these traditional biomarkers by integrating multiple hematological components that reflect both innate and adaptive immune responses [32,33,34,35,36]. This broader immunological perspective may offer enhanced prognostic value in the cardiovascular setting.

As the highest level of evidence [37,38], an umbrella review (UR) synthesizes findings from multiple systematic reviews and meta-analyses [39,40,41]. By structuring our analysis around these five categories, we aim to provide a holistic evaluation of immune-inflammatory indices in cardiovascular disease risk stratification and management. To our knowledge, this is the first UR to comprehensively assess the associations between immune-inflammatory indices and clinical outcomes in CVD patients. Our findings aim to strengthen clinical decision-making, guide future research, and improve cardiovascular risk prediction models by offering a consolidated evaluation of these biomarkers as prognostic tools. Understanding the role of immune-inflammatory indices in CVD prognosis could help refining personalized treatment approaches, optimize therapeutic interventions, and ultimately reduce the global burden of CVD.

## 2. Materials and Methods

### 2.1. Umbrella Review Methods

This UR was conducted and reported in line with the Preferred Reporting Items for Systematic Reviews and Meta-Analyses (PRISMA) checklist (Supplementary Table S1) [42]. Our protocol was prospectively registered at the PROSPERO, the International Prospective Register of Systematic Reviews (ID: CRD42025629734) (https://www.crd.york.ac.uk/PROSPERO/ accessed on 19 December 2024).

### 2.2. Search Strategy

A systematic search was conducted across six databases: PubMed, Cochrane Library, Web of Science, Embase, Scopus, and Medline, covering the period from their inception to January 2025 (last update). The search strategy was developed using a combination of Medical Subject Headings (MeSH) terms, keywords, and text variations, following the Scottish Intercollegiate Guidelines Network (SIGN) guidance for literature searching. The search included terms related to immune-inflammatory indices (e.g., NLR, SII, PLR, SIRI), a comprehensive spectrum of CVD, and study design filters (systematic review with meta-analysis). The detailed search strategy, including the full query syntax for each database, is provided in Supplementary Table S2. To ensure comprehensive coverage, the reference lists of eligible studies were manually screened for additional relevant citations. EndNote 21 software was used to remove duplicate citations, and the sieving of articles was undertaken independently by two authors (H-XL and P-WW). Disagreements were resolved by discussion and consensus with an independent senior investigator (X-LX).

### 2.3. Selection Criteria

The inclusion criteria for studies were based on the PI[E]COS (Population, Intervention or Exposure, Comparison, Outcome, Study Design) framework, as summarized in Table 1.

### 2.4. Data Extraction

Two trained authors (H-XL and P-WW) performed data extraction independently using a predefined extraction form. Data from each article was reviewed twice to verify consistency, and any discrepancies were resolved through comparison by a senior investigator (X-LX) to ensure the final dataset accurately reflects the information from the included articles. The extracted data included information on population characteristics, immune-inflammatory indices, clinical outcomes, first author and year, sample size (cases and total), meta-analysis metrics, effect estimates with 95% confidence intervals (CI), number of studies (total, cohort, and population-based case-control), effects model, measures of heterogeneity (*I*^2^ and *Q* test *p* value), and Egger’s test *p* value.

When different SRMAs evaluated the same immune-inflammatory index, population, outcome, and comparison type, potential overlap of primary studies was expected. Following the approach of Poole et al. [43], for each identical index–outcome–comparison combination, we included only one SRMA in the main synthesis, selecting the most recent study available. If two or more SRMAs were published within a comparable period for the same combination, we retained the one with the largest number of cohort studies, and if still equivalent, the highest AMSTAR-2 quality rating. This ensured that each set of primary data was represented once while maintaining completeness across outcomes.

### 2.5. Quality Assessment of Methods and Evidence

Two independent reviewers (H-XL and P-WW) used A Measurement Tool to Assess Systematic Reviews 2 (AMSTAR-2) to rate the included SRMAs, which evaluates studies across seven critical and nine noncritical domains. Each domain was rated as “yes,” “partially yes,” or “no” to indicate adherence to quality standards [44], which comprises seven critical and nine non-critical domains. Each domain was rated Yes (criterion met), Partial Yes (partially met), or No (not met). Overall confidence in the results was derived from the domain-level ratings according to AMSTAR-2 guidance: High (no or one non-critical weakness; i.e., ≤1 domain rated Partial Yes and all critical domains rated Yes), Moderate (>1 non-critical weakness with all critical domains rated Yes), Low (≥1 critical weakness; i.e., any critical domain rated No, irrespective of non-critical weaknesses), or Critically low (≥2 critical weaknesses; ≥2 critical domains rated No). Disagreements were resolved by discussion and consensus.

### 2.6. Grading the Evidence

The quality of evidence for each outcome included in the UR was assessed using the Grading of Recommendations, Assessment, Development, and Evaluation (GRADE) framework [45]. The GRADE approach evaluates the quality of evidence from SRMAs and categorizes it into four levels: high, moderate, low, or very low. The assessment was conducted independently by two authors (H-XL and P-WW), who evaluated the methodological quality and the overall quality of evidence. Any disagreements were resolved through discussion and consensus with a third independent author (X-LX). This rigorous process ensured a systematic and objective evaluation of the evidence included in the review.

### 2.7. Statistical Analysis

Data analysis was performed with Review Manager (RevMan, version 5.4) and Stata (version 18.0). We extracted various effect sizes, including odds ratios (ORs), risk ratios (RRs), hazard ratios (HRs), standardized mean differences (SMDs), mean differences (MDs), and weighted mean differences (WMDs), along with their corresponding 95% confidence intervals (CI) from the included meta-analyses. Heterogeneity among studies was assessed using the *I*^2^ statistic and Cochran’s *Q* test, with *I*^2^ > 50% or *p* < 0.10 indicating substantial heterogeneity. A random-effects model was applied in cases of high heterogeneity, while a fixed-effects model was used when *I*^2^ < 50% or *p* > 0.10. Publication bias was evaluated through visual inspection of funnel plots.

## 3. Results

### 3.1. Study Characteristics

**Figure 1:**
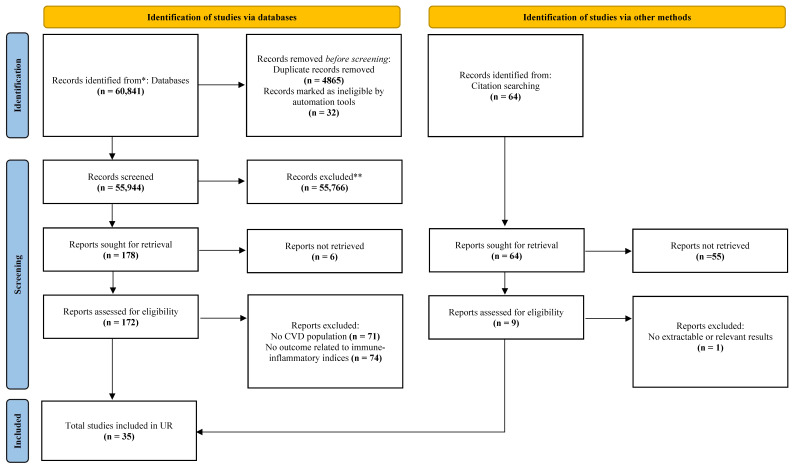
Flowchart of systematic search and selection process. Flow diagram visually summarizing the screening and selection processes, and the numbers of articles recorded at each different stage. A total of 60,841 records were identified through systematic searches of six electronic databases: PubMed (*n* = 354), Embase (*n* = 810), Web of Science (*n* = 58,351), the Cochrane Library (*n* = 595), Scopus (*n* = 333), and Medline (*n* = 398). After the removal of 4897 records, including 4865 duplicates and 32 records excluded by automated screening tools, 55,944 records remained for title and abstract screening. Among these, 55,766 records were excluded for not meeting the inclusion criteria, and the remaining 178 articles were retrieved for full-text review. Of these, 6 full-text articles were not available, and 145 were excluded for the following reasons: 71 did not involve populations with cardiovascular disease (CVD), and 74 did not assess the association between immune inflammatory indices and clinical outcomes in patients with CVD. As a result, 27 studies identified through database searches were included in the umbrella review. In addition, 64 records were identified through citation tracking. After retrieval and eligibility assessment, 8 studies met the inclusion criteria. One study was excluded due to lack of outcome data, and 55 records were not retrievable. Therefore, a total of 35 studies were included in this umbrella review, comprising 27 from database searches and 8 from citation tracking; CVD = cardiovascular disease; UR = umbrella review; * = PubMed, Cochrane Library, Web of Science, Embase, Scopus, and MEDLINE; ** = Records excluded based on title and abstract screening.

The study selection process is presented in Figure 1. A comprehensive search across multiple databases initially identified 60,841 records. After removing duplicates and screening based on title, abstract, and full text, a total of 35 studies were included in the UR [26,28,29,33,46,47,48,49,50,51,52,53,54,55,56,57,58,59,60,61,62,63,64,65,66,67,68,69,70,71,72,73,74,75,76]. Supplementary Table S3 shows the characteristics of the included Systematic Reviews and Meta-Analyses (SRMAs). This systematic selection process ensured that the included studies addressed the association between immune-inflammatory indices and clinical outcomes in patients with existing cardiovascular conditions, minimizing potential bias and enhancing the validity of the UR.

### 3.2. Quality of Included Studies

The methodological quality of the included SRMAs was evaluated using the AMSTAR-2 tool, with most studies rated as moderate or high quality. The certainty of associations was assessed using the GRADE framework, with none reaching high certainty and most rated as low or very low. Detailed results of both quality assessments are summarized below.

According to the AMSTAR-2 rating, of the 35 included systematic reviews, 15 (42.9%) were rated as high quality, 18 (51.4%) as moderate quality, and 2 (5.7%) as low quality. From these 35 studies, 106 associations were extracted, among which 87 demonstrated significant associations. Of these significant associations, 83 (95.4%) were derived from studies rated as moderate or high quality, reinforcing the credibility of our findings. The full AMSTAR-2 assessment is provided in Supplementary Table S4.

Among the 106 associations examined in this umbrella review, 87 (82.1%) demonstrated significant associations. According to the GRADE rating, none of these significant associations were classified as high-quality evidence. Specifically, 39 (44.8%) were rated as very low certainty, 27 (31.0%) as low certainty, and 21 (24.1%) as moderate certainty. The strongest evidence was observed in associations linking immune-inflammatory indices to cardiovascular mortality and major cardiovascular events, while lower-certainty evidence was more common in studies on functional outcomes and continuous variable analyses, primarily due to high heterogeneity, publication bias, and small study populations. The certainty of evidence varied across different outcome categories. Associations between immune-inflammatory indices and CVD mortality and cardiovascular events were supported by higher-certainty evidence, with a greater proportion of moderate-certainty ratings in these categories. In contrast, studies on functional outcomes and inflammatory burden were more frequently classified as low or very low certainty, largely due to greater heterogeneity, publication bias, and small sample sizes. This distribution reflects the stronger methodological robustness of mortality and major cardiovascular event studies, whereas functional and continuous variable analyses exhibited greater variability and lower precision in their findings. The complete GRADE assessment is detailed in Supplementary Table S5.

### 3.3. Association Between Immune-Inflammatory Indices and CVD Mortality

Across the included meta-analyses, mortality was defined variably, encompassing all-cause, cardiovascular, cardiac-related, short-term, long-term, and in-hospital mortality, depending on the population and analytical framework of each study. For consistency, these outcomes are collectively referred to as mortality throughout this section.

**Figure 2:**
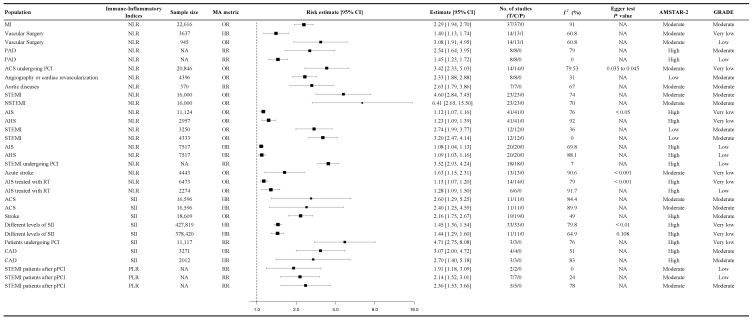
Summary random-effects estimates with 95% confidence intervals from meta-analyses of immune-inflammatory indices and mortality. This forest plot summarizes meta-analysis results on the predictive value of immune-inflammatory indices (NLR, SII, PLR) for mortality in patients with diagnosed CVD. Risk estimates are reported as OR, HR, and RR, each with 95% CIs. All estimates are from our own analysis, and effect models are random-effects unless otherwise stated. A higher NLR and SII were significantly associated with increased mortality risk. The analysis follows a random-effects model, and statistical heterogeneity quantifies variability across studies. These findings suggest that immune-inflammatory indices not only correlate with mortality risk but may also serve as prognostic markers for adverse outcomes in CVD patients, aiding in clinical risk stratification; CI = confidence interval; OR = odds ratio; HR = hazard ratio; RR = risk ratio; *I*^2^ = heterogeneity index; GRADE = Grading of Recommendations Assessment, Development, and Evaluation; AMSTAR-2 = A Measurement Tool to Assess Systematic Reviews 2; NA = not available or not applicable; T = total number of studies; C = cohort studies; P = population-based case-control and/or cross-sectional studies; MI = myocardial infarction; PAD = peripheral artery disease; ACS = acute coronary syndrome; PCI = percutaneous coronary intervention; STEMI = ST-elevation myocardial infarction; NSTEMI = non-ST-elevation myocardial infarction; AIS = acute ischemic stroke; AHS = acute hemorrhagic stroke; RT = reperfusion therapy; CAD = coronary artery disease; pPCI = primary percutaneous coronary intervention; NLR = neutrophil-to-lymphocyte ratio; SII = systemic immune-inflammation index; PLR = platelet-to-lymphocyte ratio.

Synthesizing findings from 37 studies including 22,616 patients demonstrated a significant association between elevated NLR and increased mortality in individuals with myocardial infarction (MI). NLR is a widely used immune-inflammatory index that reflects the balance between neutrophil-driven inflammation and lymphocyte-mediated immune regulation, with higher values indicating a pro-inflammatory state and impaired immune response. Specifically, patients with higher NLR levels exhibited a 2.29-fold increased risk of mortality compared to those with lower levels (OR 2.29, 95% CI 1.94 to 2.70, GRADE: Moderate, Figure 2) [47]. Among patients with peripheral artery disease, higher NLR levels were significantly associated with an increased risk of mortality (RR 2.54, 95% CI 1.64 to 3.95, GRADE: Moderate, Figure 2) [50]. A similar pattern was observed in patients with aortic diseases, where higher NLR levels were associated with a higher risk of mortality (RR 2.63, 95% CI 1.79 to 3.86, GRADE: Moderate, Figure 2) [55]. These findings were supported by moderate-certainty evidence.

Among patients with ST-elevation myocardial infarction, elevated NLR was strongly associated with mortality, with odds ratios ranging up to 4.60 (95% CI 2.84 to 7.45, GRADE: Moderate, Figure 2) [52]. In contrast, the risk was even more pronounced among patients with non-ST-elevation myocardial infarction (NSTEMI), with an odds ratio of 6.41 (95% CI 2.65 to 15.50, GRADE: Moderate, Figure 2) [57]. In STEMI patients undergoing percutaneous coronary intervention (PCI), NLR remained a significant predictor of mortality, with a reported risk ratio of 3.52 (95% CI 2.93 to 4.24, GRADE: Low, Figure 2) [64]. In AIS patients who received reperfusion therapy, both admission and post-treatment NLR levels were predictive of mortality, with post-treatment values showing a stronger association (OR 1.28, 95% CI 1.09 to 1.50, GRADE: Low, Figure 2) [67]. These findings were supported by moderate- to low-certainty evidence.

Elevated NLR levels were linked to increased mortality risk in vascular surgery patients (HR 1.40, 95% CI 1.13 to 1.74, GRADE: Very Low, Figure 2) [48]. Among patients with acute ischemic stroke (AIS), elevated NLR levels were significantly associated with increased mortality (OR 1.12, 95% CI 1.07 to 1.16, GRADE: Very Low, Figure 2) [59]. In patients with acute coronary syndrome (ACS) who underwent PCI, elevated NLR was significantly associated with increased mortality risk, as evidenced by an odds ratio of 3.42 (95% CI 2.32 to 5.03, GRADE: Very Low, Figure 2) [28]. In addition to NLR, our study found that the SII, a blood-test-derived immune-inflammatory marker integrating neutrophil, lymphocyte, and platelet counts ([neutrophil count × platelet count]/lymphocyte count) [77], is also a strong predictor of mortality in ACS patients. The hazard ratios ranged from 2.40 to 2.60 (95% CI: 1.25 to 5.25, GRADE: Moderate, Figure 2) [70], further emphasizing its prognostic significance in this population.

### 3.4. Association Between Immune-Inflammatory Indices and the Probability of Cardiovascular Events

**Figure 3:**
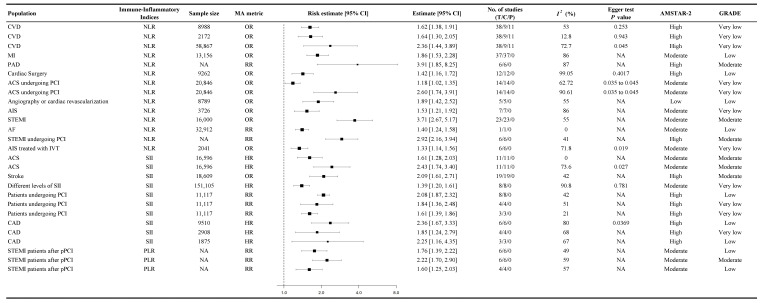
Summary random-effects estimates with 95% confidence intervals from meta-analyses of immune-inflammatory indices and cardiovascular events. This forest plot summarizes the results of meta-analyses evaluating the association between immune-inflammatory indices and cardiovascular events in patients with diagnosed CVD. The analyzed immune-inflammatory indices include the NLR, SII, and PLR. Risk estimates are presented as OR, HR, or RR, each accompanied by 95% CIs. All estimates are from our own analysis, and effect models are random-effects unless otherwise stated. The size of the markers represents the weight of each meta-analysis, and statistical heterogeneity is reported for each analysis. Findings indicate that elevated NLR and SII levels are associated with a higher risk of adverse cardiovascular events. Our results indicate that immune-inflammatory indices may help predict cardiovascular events and aid in clinical decision-making; CI = confidence interval; OR = odds ratio; HR = hazard ratio; RR = risk ratio; *I*^2^ = heterogeneity index; GRADE = Grading of Recommendations Assessment, Development, and Evaluation; AMSTAR-2 = A Measurement Tool to Assess Systematic Reviews 2; NA = not available or not applicable; T = total number of studies; C = cohort studies; P = population-based case-control and/or cross-sectional studies; CVD = cardiovascular disease; MI = myocardial infarction; PAD = peripheral artery disease; ACS = acute coronary syndrome; PCI = percutaneous coronary intervention; AIS = acute ischemic stroke; STEMI = ST-elevation myocardial infarction; AF = atrial fibrillation; IVT = intravenous thrombolysis; CAD = coronary artery disease; pPCI = primary percutaneous coronary intervention; NLR = neutrophil-to-lymphocyte ratio; SII = systemic immune-inflammation index; PLR = platelet-to-lymphocyte ratio.

Across multiple analyses, NLR was found to be significantly associated with an increased probability of cardiovascular events. In a pooled analysis of 38 studies involving 8988 patients, higher NLR levels were linked to an increased likelihood of experiencing cardiovascular events (OR 1.62, 95% CI 1.38 to 1.91, GRADE: Very Low, Figure 3) [46]. An expanded dataset including 58,867 patients confirmed this association with a more substantial effect size (OR 2.36, 95% CI 1.44 to 3.89, GRADE: Very Low, Figure 3) [46].

In patients with STEMI undergoing PCI, elevated NLR was associated with a significantly increased risk of cardiovascular events, with a risk ratio of 2.92 (95% CI 2.16 to 3.94, GRADE: Moderate, Figure 3) [64]. Among ACS patients undergoing PCI, the relationship between NLR and cardiovascular events was complex, with odds ratios ranging from 1.175 (95% CI 1.021 to 1.353, GRADE: Very Low, Figure 3) to 2.604 (95% CI 1.736 to 3.906, GRADE: Very Low, Figure 3) [28].

In AIS patients, NLR was significantly associated with a higher probability of cardiovascular events (OR 1.53, 95% CI 1.21 to 1.92, GRADE: Very Low, Figure 3) [56]. Additionally, higher SII levels were found to be linked to an increased risk of cardiovascular events in stroke patients (OR 2.09, 95% CI 1.61 to 2.71, GRADE: Moderate, Figure 3) [68]. These findings were supported by low- to moderate-certainty evidence.

### 3.5. Association Between Immune-Inflammatory Indices and Functional Outcomes

**Figure 4:**
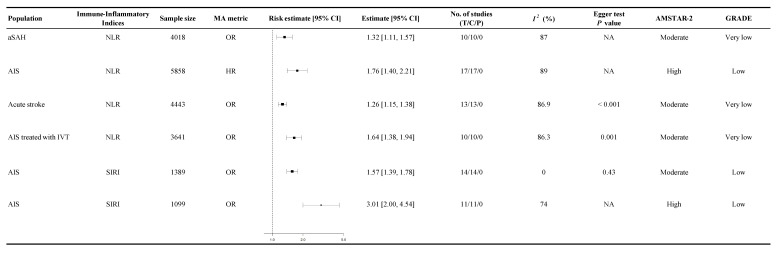
Summary random-effects estimates with 95% confidence intervals from meta-analyses of immune-inflammatory indices and functional outcomes. This forest plot presents summary estimates from meta-analyses evaluating the association between immune-inflammatory indices and functional outcomes in patients with acute cerebrovascular diseases, including aSAH and AIS. The analyzed immune-inflammatory indices include the NLR and SIRI. Risk estimates are reported as OR and HR, each accompanied by 95% CIs. All estimates are from our own analysis, and effect models are random-effects unless otherwise stated. The statistical heterogeneity index quantifies variability across studies. Elevated NLR and SIRI were significantly associated with poorer functional outcomes, suggesting their role in stroke prognosis; CI = confidence interval; OR = odds ratio; HR = hazard ratio; *I*^2^ = heterogeneity index; GRADE = Grading of Recommendations Assessment, Development, and Evaluation; AMSTAR-2 = A Measurement Tool to Assess Systematic Reviews 2; NA = not available or not applicable; T = total number of studies; C = cohort studies; P = population-based Case–Control and/or cross-sectional studies; aSAH = aneurysmal subarachnoid hemorrhage; AIS = acute ischemic stroke; IVT = intravenous thrombolysis; NLR = neutrophil-to-lymphocyte ratio; SIRI = systemic inflammatory response index.

Functional outcomes generally reflected patients’ post-event functional status or degree of disability, assessed through validated clinical measures commonly including the modified Rankin Scale, Glasgow Outcome Scale, or comparable evaluations. Among AIS patients who received intravenous thrombolysis (IVT), higher NLR levels were associated with an increased risk of poor functional outcomes (OR 1.64, 95% CI 1.38 to 1.94, GRADE: Very Low, Figure 4) [66]. SIRI is another composite inflammatory index incorporating monocyte, neutrophil, and lymphocyte counts, reflecting innate immune activation and adaptive immune suppression. In addition to NLR, the SIRI was also strongly associated with poor functional outcomes in AIS patients, with odds ratios ranging from 1.57 (95% CI 1.39 to 1.78, GRADE: Low, Figure 4) to 3.01 (95% CI 2.00 to 4.54, GRADE: Low, Figure 4) [29,74]. These findings were supported by low- to very-low-certainty evidence.

### 3.6. Association Between Immune-Inflammatory Indices and Other Cardiovascular-Related Conditions

**Figure 5:**
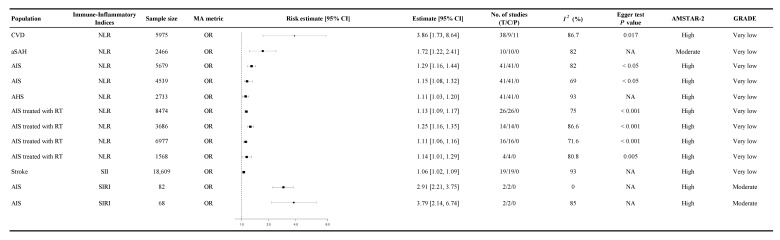
Summary random-effects estimates with 95% confidence intervals from meta-analyses of immune-inflammatory indices and other cardiovascular-related conditions. This forest plot presents summary estimates from meta-analyses assessing the association between immune-inflammatory indices and various cardiovascular-related conditions, including CVD, AIS, aSAH, and stroke. The analyzed immune-inflammatory indices include the NLR, SII, and SIRI. Risk estimates are reported as OR and HR, each with 95% CIs. All estimates are from our own analysis, and effect models are random-effects unless otherwise stated. Statistical heterogeneity is reported for each analysis. These findings reinforce the association between immune-inflammatory indices and a broad range of cardiovascular conditions, suggesting their potential role in comprehensive cardiovascular risk assessment; CI = confidence interval; OR = odds ratio; *I*^2^ = heterogeneity index; GRADE = Grading of Recommendations Assessment, Development, and Evaluation; AMSTAR-2 = A Measurement Tool to Assess Systematic Reviews 2; NA = not available or not applicable; T = total number of studies; C = cohort studies; P = population-based case-control and/or cross-sectional studies; CVD = cardiovascular disease; aSAH = aneurysmal subarachnoid hemorrhage; AIS = acute ischemic stroke; AHS = acute hemorrhagic stroke; RT = reperfusion therapy; NLR = neutrophil-to-lymphocyte ratio; SII = systemic immune-inflammation index; SIRI = systemic inflammatory response index.

A meta-analysis including 5975 patients with CVD demonstrated that elevated NLR levels were associated with a significantly increased risk of composite cardiovascular outcomes, with an odds ratio of 3.86 (95% CI 1.73 to 8.64, GRADE: Very Low, Figure 5) [46]. Similarly, in patients with aneurysmal subarachnoid hemorrhage (aSAH), increased NLR levels were linked to a higher likelihood of developing delayed cerebral ischemia (DCI), with an odds ratio of 1.72 (95% CI 1.22 to 2.41, GRADE: Very Low, Figure 5) [53]. Among patients with AIS, higher NLR levels were associated with poor outcomes (OR 1.29, 95% CI 1.16 to 1.44, GRADE: Very Low, Figure 5) [59].

The SIRI also showed a strong predictive value for adverse neurological outcomes. In AIS patients, higher SIRI levels were associated with an increased risk of stroke-associated pneumonia (SAP) (OR 2.91, 95% CI 2.21 to 3.75, GRADE: Moderate, Figure 5) and early neurological deterioration (END) (OR 3.79, 95% CI 2.14 to 6.74, GRADE: Moderate, Figure 5) [29].

### 3.7. Inflammatory Burden

**Figure 6:**
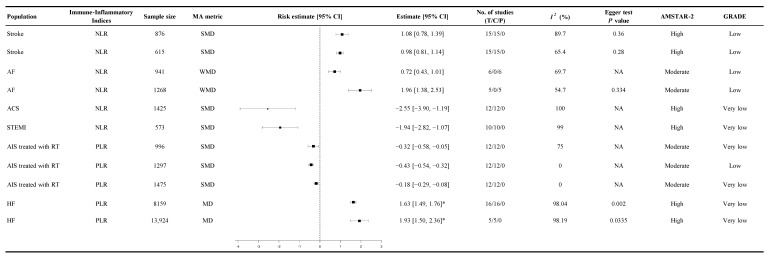
Summary random-effects estimate 95% confidence intervals from meta-analyses of inflammatory burden and cardiovascular outcomes. This forest plot presents summary estimates from meta-analyses evaluating the relationship between immune-inflammatory indices and continuous cardiovascular outcomes, including stroke, AF, ACS, STEMI, HF, and AIS treated with RT. The analyzed immune-inflammatory indices include the NLR and PLR. Risk estimates are expressed as SMD, WMD, or MD, each with corresponding 95% CIs. All estimates are derived from our own analysis, and effect models are random-effects unless otherwise specified. Statistical heterogeneity is reported for each analysis. Higher NLR and PLR values were associated with greater systemic inflammation and adverse changes in cardiovascular function. These associations suggest that immune-inflammatory indices may reflect the severity of cardiovascular disease and provide insight into the inflammatory burden influencing disease progression and treatment outcomes; CI = confidence interval; SMD = standardized mean difference; WMD = weighted mean difference; MD = mean difference; *I*^2^ = heterogeneity index; GRADE = Grading of Recommendations Assessment, Development, and Evaluation; AMSTAR-2 = A Measurement Tool to Assess Systematic Reviews 2; NA = not available or not applicable; T = total number of studies; C = cohort studies; P = population-based case-control and/or cross-sectional studies; AF = atrial fibrillation; ACS = acute coronary syndrome; STEMI = ST-elevation myocardial infarction; AIS = acute ischemic stroke; RT = reperfusion therapy; HF = heart failure; NLR = neutrophil-to-lymphocyte ratio; PLR = platelet-to-lymphocyte ratio; * ×100.

Inflammatory burden refers to continuous immune-inflammatory indices such as NLR, PLR, SIRI, or SII measured on a continuous scale. In stroke patients, higher NLR levels were modestly associated with poor outcomes, with SMD ranging from 1.08 (95% CI 0.78 to 1.39, GRADE: Low, Figure 6) to 0.98 (95% CI 0.81 to 1.14, GRADE: Low, Figure 6) [49]. PLR is an inflammatory biomarker that reflects the balance between platelet activation and lymphocyte-mediated immune response. Among AIS patients treated with RT, higher PLR at admission was associated with worse 90-day functional recovery, referring to continuous measures of post-stroke functional status (SMD −0.32, 95% CI −0.58 to −0.05, GRADE: Very Low, Figure 6) [75]. Delayed PLR values demonstrated a slightly stronger association with poor functional recovery (SMD −0.43, 95% CI −0.54 to −0.32, GRADE: Low, Figure 6) [75]. In HF patients, PLR was significantly correlated with increased mortality, with follow-up data indicating a mean difference of 162.55 (95% CI 149.35 to 175.75, GRADE: Very Low, Figure 6) [26]. These findings were supported by low- to very-low-certainty evidence.

### 3.8. Heterogeneity

A total of 87 sets of data reported the heterogeneity index (*I*^2^). Among these, 67 datasets showed significant heterogeneity, generally characterized by *I*^2^ values equal to or greater than 50% and corresponding *Q*-test *p*-values less than 0.05. The high proportion of studies with significant heterogeneity implies that multiple factors might influence the consistency of results when exploring the relationship between immune-inflammatory indices and various health outcomes. These factors could include differences in patient demographics, study designs, measurement methods of immune-inflammatory index, and the specific clinical settings of the included populations. Such heterogeneity should be carefully considered when interpreting the overall associations and may necessitate further research to understand better and control these confounding elements.

### 3.9. Assessment of Risk of Bias

A total of 31 studies reported the Egger’s test results. Among them, 22 studies showed evidence of publication bias (Egger’s test *p* < 0.05). These included HF studies using the PLR indicator (*p* values of 0.002 and 0.0335), Acute stroke and AIS treated with IVT using the NLR indicator (*p* < 0.001 and *p* = 0.001, respectively), CVD with the NLR indicator (*p* = 0.017 and 0.045), AIS with the NLR indicator in multiple analyses (*p* < 0.05 and *p* < 0.001), AIS treated with RT with Admission NLR and post-treatment NLR (*p* < 0.001 and *p* = 0.005), ACS undergoing PCI with the NLR indicator (*p* = 0.035–0.045), AIS treated with IVT with the NLR indicator (*p* = 0.019), ACS with the SII indicator (*p* = 0.027), and CAD with the SII indicator (*p* = 0.0369). On the other hand, 9 studies showed no significant publication bias (Egger’s test *p* > 0.05). These were Stroke with the NLR indicator (*p* values of 0.36 and 0.28), AF with the NLR indicator (*p* = 0.334), AIS with the SIRI indicator (*p* = 0.43), Different levels of SII (*p* = 0.108), CVD with the NLR indicator in multiple analyses (*p* = 0.253 and 0.943), Cardiac Surgery with Preoperative NLR (*p* = 0.4017), and Different levels of SII when evaluating CVD risk (*p* = 0.781). These findings suggest that the results of the studies with publication bias may be influenced, and caution should be exercised when interpreting them.

## 4. Discussion

This umbrella review synthesizes evidence from published systematic reviews and meta-analyses to evaluate the predictive value of immune-inflammatory indices for clinical outcomes in CVD. We analyzed 35 meta-analyses encompassing 106 unique outcomes, of which 87 demonstrated statistically significant associations. To our knowledge, this is the first comprehensive evaluation of the relationship between immune-inflammatory indices (NLR, SII, PLR, and SIRI) and clinical outcomes of patients who were diagnosed with CVD. This study followed a systematic approach, including independent literature screening, study selection, and data extraction by two authors. When sufficient data were available, we extracted reported effect sizes (RR, HR, OR, WMD, SMD or MD) with 95% CI using random or fixed-effects models. We also assessed heterogeneity and publication bias in each including meta-analysis. Overall, our findings indicate that elevated immune-inflammatory indices (NLR, SII, PLR, and SIRI) are consistently associated with adverse clinical outcomes in patients diagnosed with CVD, underscoring their potential utility as prognostic biomarkers for risk stratification and clinical management.

### 4.1. Neutrophil-to-Lymphocyte Ratio (NLR)

Our findings indicate that elevated NLR is consistently associated with increased CVD mortality and adverse cardiovascular events. The NLR is an emerging inflammatory marker derived from routine peripheral blood cell counts. In recent years, it has been widely used to assess the risk and prognosis of various chronic diseases, including CVD [78,79]. Our findings are consistent with previous research [80,81]. A retrospective cohort study from China demonstrated a positive correlation between elevated NLR levels and increased mortality in MI patients [80]. Furthermore, a large-scale cohort study reported that higher NLR levels were significantly associated with various cardiovascular outcomes, including CAD, ACS, stroke, and composite cardiovascular events [81].

Clinically, NLR could aid in risk stratification, particularly in patients undergoing revascularization procedures, where heightened inflammatory responses may predict worse outcomes [82,83]. However, its prognostic value may be affected by acute infections, malignancies, or other systemic inflammatory conditions that transiently alter neutrophil and lymphocyte counts [84,85]. Further research is needed to explore its potential role in guiding anti-inflammatory and immunomodulatory therapies in CVD patients.

### 4.2. Systemic Immune-Inflammation Index (SII)

Our findings demonstrated that elevated SII was strongly associated with adverse cardiovascular outcomes, particularly in patients with ACS and CAD. Among patients undergoing PCI, higher SII levels were predictive of increased mortality. Moreover, SII was significantly linked to stroke outcomes, highlighting its prognostic utility beyond coronary syndromes. These findings align with prior evidence supporting the prognostic value of SII in cardiovascular health [86,87]. A prospective cohort study in China found that elevated SII was associated with an increased risk of total stroke and its subtypes, reinforcing its role in cerebrovascular risk stratification [86]. Additionally, a large-scale study involving 85,154 individuals with a 10-year follow-up confirmed that higher SII levels significantly increased the risk of CVD and ACM [87]. Further validation by Xia strengthened the link between SII and adverse cardiovascular outcomes, highlighting its reliability as a prognostic indicator across various cardiovascular conditions, including coronary and cerebrovascular diseases [87].

The inclusion of platelet count makes SII particularly relevant in conditions with a pronounced thrombo-inflammatory component, such as acute coronary events and stroke [88,89]. However, variations in platelet function and reactivity among individuals, particularly in patients receiving antiplatelet therapy, may affect its predictive accuracy. Future research should assess the role of SII in guiding antithrombotic strategies and its potential integration into risk prediction models for thrombosis-related complications.

### 4.3. Platelet-to-Lymphocyte Ratio (PLR)

Our result shows that higher PLR levels were significantly associated with worse functional recovery in stroke patients undergoing RT. Additionally, among STEMI patients undergoing PCI, elevated PLR was linked to an increased risk of mortality, a result that corroborates Willim’s findings [90]. Specifically, Willim et al. identified PLR as a predictive marker for both in-hospital and long-term adverse outcomes in STEMI patients undergoing primary PCI, suggesting its potential utility for risk stratification and guiding clinical management [90]. They also noted variability in optimal cutoff values across studies, highlighting the need for further research to establish standardized thresholds for clinical application [90].

One potential advantage of PLR over NLR and SII is its ability to reflect the balance between prothrombotic and immunoregulatory pathways [91]. However, given that platelet counts are influenced by multiple factors, including medications [92], nutritional status [93], and hematological disorders [94], careful interpretation is required when using PLR in clinical decision-making. Further studies should explore whether PLR can personalize antiplatelet therapy in CVD patients.

### 4.4. Systemic Inflammatory Response Index (SIRI)

Our study found that elevated SIRI is strongly associated with poor prognosis in acute ischemic stroke, including higher mortality and impaired functional outcomes. This aligns with previous animal and clinical studies, which have demonstrated that focal ischemia and hypoxia in the acute phase of stroke trigger a cascade of immune responses, characterized by the recruitment of peripheral immune cells and secretion of inflammatory cytokines [95,96,97].

These inflammatory mediators can persist in the affected brain regions for weeks, contributing to secondary brain injury and worsening patient outcomes [98]. SIRI’s inclusion of monocytes makes it particularly relevant in conditions characterized by chronic inflammation and immune activation [99]. However, its clinical applicability remains limited by variability in monocyte response under different disease states [100]. Future research should assess whether SIRI can predict response to anti-inflammatory therapies and guide individualized treatment strategies in stroke and cardiovascular patients.

### 4.5. Strengths

Our study comprehensively synthesizes existing evidence on the relationship between immune-inflammatory indices and clinical outcomes of patients with CVD, providing valuable insights for clinical practice and future research. One of its key strengths lies in its rigorous methodological approach. We conducted a systematic search across multiple scientific databases, including all relevant systematic reviews with meta-analyses. To minimize potential bias and enhance reliability, study selection and data extraction were performed independently by trained researchers. Additionally, we employed two standardized assessment tools, AMSTAR-2 and GRADE, to evaluate the methodological quality and strength of evidence. Notably, 102 of the 106 associations (96.2%) included in this review were rated as moderate to high quality based on AMSTAR-2, reinforcing the credibility of our findings. Another major strength of this study is the comprehensive evaluation of multiple immune-inflammatory indices, including the NLR, SII, PLR and SIRI. By systematically assessing their predictive value across various cardiovascular outcomes (such as MI, stroke, HF, and ACM). This review enhances the clinical applicability of these biomarkers and provides a theoretical foundation for future risk stratification and personalized management.

### 4.6. Limitations

Despite its strengths, this study has several important limitations. First, as an umbrella review, its findings depend on the quality and completeness of existing systematic reviews with meta-analyses. Substantial heterogeneity was observed across most associations, and according to GRADE, none of the outcomes reached high certainty, with the majority rated as low or very low. Such variability likely reflects differences in study populations, thresholds used to define inflammatory indices, and analytic approaches. These inconsistencies may reduce the precision and generalizability of the conclusions. Although some studies adjusted for conventional cardiovascular risk factors, variation in adjustment strategies across meta-analyses raises the possibility of residual bias.

Second, the absence of standardized cutoffs across the included meta-analyses precluded formal dose–response evaluation and hindered assessment of whether incrementally higher index levels translate into stepwise increases in risk. Definitions of “high” versus “low” were heterogeneous or unreported, reflecting the use of cohort-specific thresholds, ROC-derived dichotomies, or continuous modeling approaches. This methodological variability substantially reduces comparability across studies and complicates interpretation of pooled estimates. Moreover, most source studies evaluated these indices at a single time point, without capturing longitudinal changes that might offer deeper prognostic insight. This limitation underscores the lack of evidence on temporal dynamics in inflammatory activity and their potential prognostic implications.

Third, the available evidence has primarily focused on major cardiovascular outcomes, while preclinical markers such as endothelial dysfunction, arterial stiffness, and subclinical organ damage remain largely unexplored. The predominance of observational studies introduces vulnerability to unmeasured confounding and publication bias. Much of the evidence also derives from Asian cohorts, particularly from China, which restricts the applicability of findings to other populations. Few analyses evaluated whether these indices provide incremental value beyond established risk models such as the Framingham score, the Global Registry of Acute Coronary Events score, or the CHA_2_DS_2_-VASc score. Practical barriers, including limited clinician awareness, inconsistent reporting in laboratory systems, and lack of regulatory frameworks, were also rarely addressed. Finally, although we applied Egger’s test and related methods to assess small-study effects, the influence of unpublished null findings or selective reporting cannot be excluded.

### 4.7. Future Directions

**Figure 7:**
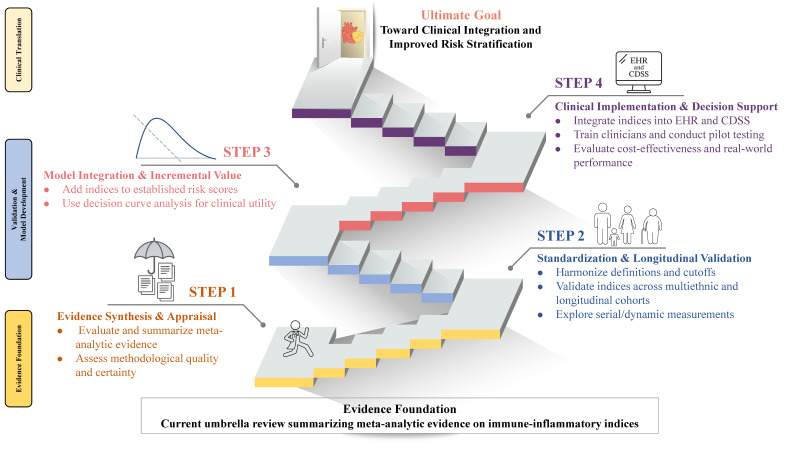
Proposed translational roadmap for integrating immune-inflammatory indices into cardiovascular risk stratification and clinical practice. Conceptual roadmap showing the phased advancement of immune-inflammatory indices (neutrophil-to-lymphocyte ratio [NLR], systemic immune-inflammation index [SII], platelet-to-lymphocyte ratio [PLR], and systemic inflammation response index [SIRI]) toward cardiovascular risk stratification and clinical use. The left panel groups activities into three phases: Phase I, Evidence Foundation (Step 1: evidence synthesis and appraisal); Phase II, Validation and Model Development (Steps 2–3: standardization and longitudinal validation, followed by model integration with assessment of incremental value); and Phase III, Clinical Translation (Step 4: implementation within electronic health records and clinical decision support systems, clinician training, and real-world evaluation). Our umbrella review maps to Phase I. This schema is intended as a high-level framework summarizing domains of ongoing and future work rather than a prescriptive sequence or clinical guidance; EHR = electronic health record; CDSS = clinical decision support system.

Future research should prioritize the development of harmonized definitions and cutoff values and incorporate longitudinal designs to capture dynamic changes in immune-inflammatory indices. In particular, prospective studies incorporating dynamic biomarkers or serial measurements are needed to better characterize temporal variations and their prognostic significance. Expanding investigations to preclinical outcomes that may precede overt cardiovascular disease will further elucidate underlying mechanisms. Multiethnic cohorts are essential to enhance generalizability, and comparative studies should test whether these indices improve predictive performance beyond established risk models. Equally important, translational efforts must address practical barriers including laboratory standardization, clinician training, and regulatory guidance to facilitate the integration of immune-inflammatory indices into cardiovascular risk assessment and clinical practice. A proposed research roadmap for clinical translation of these indices is illustrated in Figure 7.

## 5. Conclusions

Our findings indicate that elevated NLR, SII, PLR, and SIRI are significantly associated with higher mortality, increased adverse cardiovascular events, and worse functional outcomes. Among these indices, NLR and SII consistently showed robust predictive value across multiple cardiovascular conditions, whereas PLR and SIRI were particularly linked to poor prognosis in stroke populations. Despite these promising associations, clinical translation is hindered by the absence of standardized cutoffs, limited validation in multiethnic cohorts, and insufficient integration with established risk models. Future research should therefore prioritize harmonizing definitions, testing incremental value beyond existing scores, and validating prognostic accuracy across diverse populations. Collectively, our umbrella review provides an evidence base for incorporating immune-inflammatory indices into cardiovascular risk prediction frameworks, bridging the gap between observational evidence and clinical translation to enhance patient management and outcomes.

## Figures and Tables

**Table 1:** Study Inclusion and Exclusion Criteria.

Category	Inclusion Criteria
**Population**	Patients with a broad range of CVD, including but not limited to CAD, CeVD (e.g., stroke), PAD, HF, CMPs, and other cardiac conditions
**Exposure**	At least one immune-inflammatory index (NLR, PLR, SII, SIRI)
**Comparison**	Low vs. high levels of immune-inflammatory indices
**Outcome**	Clinical outcomes of CVD patients, such as mortality, MACE, hospitalization, revascularization, recurrence rates, and other related outcomes
**Study design**	Systematic reviews with meta-analyses containing quantitative data
**Language**	English

Summary of the study framework based on the PI[E]COS approach. This table outlines the inclusion criteria for study selection, focusing on cardiovascular disease populations, immune-inflammatory indices (NLR, PLR, SII, SIRI), and clinically relevant outcomes such as mortality, MACE, hospitalization, and revascularization. Only systematic reviews with meta-analyses reporting quantitative data were included. Studies not meeting these criteria were excluded; CVD = cardiovascular disease; CeVD = Cerebrovascular disease; CAD = coronary artery disease; PAD = peripheral arterial disease; HF = heart failure; CMPs = cardiomyopathies; NLR = neutrophil-to-lymphocyte ratio; PLR = platelet-to-lymphocyte ratio; SII = systemic immune-inflammation index; SIRI = systemic inflammatory response index; vs. = versus; MACE = major adverse cardiovascular events.

## Data Availability

The original contributions presented in this study are included in the article, and further inquiries can be directed to the corresponding author.

## Supplementary Materials

The following supporting information can be downloaded at https://www.mdpi.com/article/10.3390/diagnostics15222862/s1, Supplementary Table S1: PRISMA 2020 Checklist; Supplementary Table S2: Search Terms and Search Strategies for Six Databases; Supplementary Table S3: Characteristics of Included Systematic Reviews and Meta-Analyses; Supplementary Table S4: Assessments of AMSTAR-2 Scores; Supplementary Table S5: GRADE Classification of Quality of Evidence. Reference [101] is cited in the Supplementary Materials.

## Abbreviations

The following abbreviations are used in this manuscript:

ACS	acute coronary syndrome
ACM	all-cause mortality
AF	atrial fibrillation
AIS	acute ischemic stroke
AHS	acute hemorrhagic stroke
AMSTAR-2	A Measurement Tool to Assess Systematic Reviews 2
aSAH	aneurysmal subarachnoid hemorrhage
CAD	coronary artery disease
CDSS	clinical decision support system
CeVD	cerebrovascular disease
CRP	C-reactive protein
CI	confidence interval
CMPs	cardiomyopathies
CVD	cardiovascular disease
DALYs	disability-adjusted life years
DCI	delayed cerebral ischemia
EHR	electronic health record
END	early neurological deterioration
GRADE	Grading of Recommendations Assessment, Development, and Evaluation
HF	heart failure
HR	hazard ratio
*I* ^2^	inconsistency index (statistical measure of heterogeneity)
IVT	intravenous thrombolysis
MACE	major adverse cardiovascular events
MD	mean difference
MI	myocardial infarction
MeSH	Medical Subject Headings
NA	not available or not applicable
NCD	non-communicable disease
NLR	neutrophil-to-lymphocyte ratio
NSTEMI	non-ST-elevation myocardial infarction
OR	odds ratio
PAD	peripheral artery disease
PCI	percutaneous coronary intervention
pPCI	primary percutaneous coronary intervention
PLR	platelet-to-lymphocyte ratio
RR	risk ratio
PRISMA	Preferred Reporting Items for Systematic Reviews and Meta-Analyses
PROSPERO	International Prospective Register of Systematic Reviews
PI[E]COS	Population, Intervention/Exposure, Comparison, Outcome, Study design
RT	reperfusion therapy
SAP	stroke-associated pneumonia
SII	systemic immune-inflammation index
SIRI	systemic inflammation response index
SIGN	Scottish Intercollegiate Guidelines Network
SMD	standardized mean difference
STEMI	ST-elevation myocardial infarction
SRMA	systematic review and meta-analysis
UR	umbrella review
vs.	versus
WHO	World Health Organization
WMD	weighted mean difference
