# Supplementary material for: Evaluating Immune-Inflammatory Indices for Risk Stratification in Cardiovascular Disease: An Umbrella Review of Systematic Reviews and Meta-Analyses
Source: Diagnostics (Basel). 2025 Nov 12;15(22):2862. doi: 10.3390/diagnostics15222862 (PMC12651550; doi:10.3390/diagnostics15222862)
Supplement: Supplementary file 1 [file diagnostics-15-02862-s001.zip › diagnostics-3924545-supplementary.pdf]

SUPPLEMENTARY MATERIALS FOR:

"Evaluating Immune-Inflammatory Indices for Risk Stratification in Cardiovascular Disease: An Umbrella Review of Systematic Reviews and Meta-Analyses"

Table of Contents

1. Supplementary Table S1 PRISMA 2020 Checklist 2

2. Supplementary Table S2 Search Terms and Search Strategies for Six Databases 7

3. Supplementary Table S3 Characteristics of Included Systematic Reviews and Meta-Analyses 11

4. Supplementary Table S4 Assessments of AMSTAR-2 Scores 29

5. Supplementary Table S5 GRADE Classification of Quality of Evidence 33

6. Supplementary References 58

1. Supplementary Table S1 PRISMA 2020 Checklist

Section and Topic	Item #	Checklist item	Location where item is reported
**TITLE**			
Title	1	Identify the report as a systematic review.	Title
**ABSTRACT**			
Abstract	2	See the PRISMA 2020 for Abstracts checklist.	Abstract
**INTRODUCTION**			
Rationale	3	Describe the rationale for the review in the context of existing knowledge.	Introduction
Objectives	4	Provide an explicit statement of the objective(s) or question(s) the review addresses.	Introduction
**METHODS**			
Eligibility criteria	5	Specify the inclusion and exclusion criteria for the review and how studies were grouped for the syntheses.	Selection Criteria
Information sources	6	Specify all databases, registers, websites, organisations, reference lists and other sources searched or consulted to identify studies. Specify the date when each source was last searched or consulted.	Search Strategy
Search strategy	7	Present the full search strategies for all databases, registers and websites, including any filters and limits used.	Supplementary Table S2
Selection process	8	Specify the methods used to decide whether a study met the inclusion criteria of the review, including how many reviewers screened each record and each report retrieved, whether they worked independently, and if applicable, details of automation tools used in the process.	Search Strategy
Data collection process	9	Specify the methods used to collect data from reports, including how many reviewers collected data from each report, whether they worked independently, any processes for obtaining or confirming data from study investigators, and if applicable, details of automation tools used in the process.	Data Extraction
Data items	10a	List and define all outcomes for which data were sought. Specify whether all results that were compatible with each outcome domain in each study	Data Extraction

Section and Topic	Item #	Checklist item	Location where item is reported
		were sought (e.g. for all measures, time points, analyses), and if not, the methods used to decide which results to collect.	
	10b	List and define all other variables for which data were sought (e.g. participant and intervention characteristics, funding sources). Describe any assumptions made about any missing or unclear information.	Data Extraction
Study risk of bias assessment	11	Specify the methods used to assess risk of bias in the included studies, including details of the tool(s) used, how many reviewers assessed each study and whether they worked independently, and if applicable, details of automation tools used in the process.	Quality Assessment of Methods and Evidence
Effect measures	12	Specify for each outcome the effect measure(s) (e.g. risk ratio, mean difference) used in the synthesis or presentation of results.	Statistical Analysis
Synthesis methods	13a	Describe the processes used to decide which studies were eligible for each synthesis (e.g. tabulating the study intervention characteristics and comparing against the planned groups for each synthesis (item #5)).	Selection Criteria
	13b	Describe any methods required to prepare the data for presentation or synthesis, such as handling of missing summary statistics, or data conversions.	Statistical Analysis
	13c	Describe any methods used to tabulate or visually display results of individual studies and syntheses.	Results + Figures 2–6, Supplementary Tables S3–5
	13d	Describe any methods used to synthesize results and provide a rationale for the choice(s). If meta-analysis was performed, describe the model(s), method(s) to identify the presence and extent of statistical heterogeneity, and software package(s) used.	Statistical Analysis
	13e	Describe any methods used to explore possible causes of heterogeneity among study results (e.g. subgroup analysis, meta-regression).	Heterogeneity
	13f	Describe any sensitivity analyses conducted to assess robustness of the synthesized results.	Supplementary Table S5
Reporting bias assessment	14	Describe any methods used to assess risk of bias due to missing results in a synthesis (arising from reporting biases).	Assessment of Risk of Bias
Certainty	15	Describe any methods used to assess certainty (or confidence) in the body of evidence for an outcome.	Grading the Evidence +

Section and Topic	Item #	Checklist item	Location where item is reported
assessment			Supplementary Table S5
**RESULTS**			
Study selection	16a	Describe the results of the search and selection process, from the number of records identified in the search to the number of studies included in the review, ideally using a flow diagram.	Study Characteristics + Figure 1
	16b	Cite studies that might appear to meet the inclusion criteria, but which were excluded, and explain why they were excluded.	Supplementary Table S3
Study characteristics	17	Cite each included study and present its characteristics.	Supplementary Table S3
Risk of bias in studies	18	Present assessments of risk of bias for each included study.	Supplementary Table S4
Results of individual studies	19	For all outcomes, present, for each study: (a) summary statistics for each group (where appropriate) and (b) an effect estimate and its precision (e.g. confidence/credible interval), ideally using structured tables or plots.	Results + Figures 2–6
Results of syntheses	20a	For each synthesis, briefly summarise the characteristics and risk of bias among contributing studies.	Quality of Included Studies
	20b	Present results of all statistical syntheses conducted. If meta-analysis was done, present for each the summary estimate and its precision (e.g. confidence/credible interval) and measures of statistical heterogeneity. If comparing groups, describe the direction of the effect.	Results + Figures
	20c	Present results of all investigations of possible causes of heterogeneity among study results.	Heterogeneity
	20d	Present results of all sensitivity analyses conducted to assess the robustness of the synthesized results.	Supplementary Table S5
Reporting biases	21	Present assessments of risk of bias due to missing results (arising from reporting biases) for each synthesis assessed.	Assessment of Risk of Bias

Section and Topic	Item #	Checklist item	Location where item is reported
Certainty of evidence	22	Present assessments of certainty (or confidence) in the body of evidence for each outcome assessed.	Quality of Included Studies + Supplementary Table S5
**DISCUSSION**			
Discussion	23a	Provide a general interpretation of the results in the context of other evidence.	Discussion
	23b	Discuss any limitations of the evidence included in the review.	Strengths and Limitations
	23c	Discuss any limitations of the review processes used.	Strengths and Limitations
	23d	Discuss implications of the results for practice, policy, and future research.	Strengths and Limitations
**OTHER INFORMATION**			
Registration and protocol	24a	Provide registration information for the review, including register name and registration number, or state that the review was not registered.	Umbrella Review Methods
	24b	Indicate where the review protocol can be accessed, or state that a protocol was not prepared.	Umbrella Review Methods
	24c	Describe and explain any amendments to information provided at registration or in the protocol.	Umbrella Review Methods
Support	25	Describe sources of financial or non-financial support for the review, and the role of the funders or sponsors in the review.	Acknowledgements

Section and Topic	Item #	Checklist item	Location where item is reported
Competing interests	26	Declare any competing interests of review authors.	Conflict of Interest Statement
Availability of data, code and other materials	27	Report which of the following are publicly available and where they can be found: template data collection forms; data extracted from included studies; data used for all analyses; analytic code; any other materials used in the review.	Supplementary Materials section

From: Page MJ, McKenzie JE, Bossuyt PM, Boutron I, Hoffmann TC, Mulrow CD, et al. The PRISMA 2020 statement: an updated guideline for reporting systematic reviews. BMJ 2021;372:n71. doi: 10.1136/bmj.n71.

This work is licensed under CC BY 4.0. To view a copy of this license, visit https://creativecommons.org/licenses/by/4.0/

2. Supplementary Table S2 Search Terms and Search Strategies for Six Databases

Database	Search Term	Result
PubMed	#1: (((("Lymphocytes"[Mesh]) OR (((Lymphocyte) OR (Lymphoid Cells)) OR (Lymphoid Cell))) AND (ratio OR index)) OR ((((systemic immune inflammation index) OR (SII)) OR (systemic inflammation response index)) OR (SIRI)))	95,313
	#2: ((("Cardiovascular Diseases"[Mesh]) OR ((((((((((((((((((((((((((((MACE) OR (CVD)) OR (ASCVD)) OR (cardiovascular mortality)) OR (cardiovascular death)) OR (stroke)) OR (IS)) OR (coronary)) OR (heart disease)) OR (CHD)) OR (AMI)) OR (coronary artery disease)) OR (CAD)) OR (ischemic heart disease)) OR (IHD)) OR (myocardial infarction)) OR (MI)) OR (unstable angina)) OR (UA)) OR (heart failure)) OR (HF)) OR (Cardiovascular Diseases)) OR (Cardiovascular Disease)) OR (Cardiac Event)) OR (Cardiac Events)) OR (Adverse Cardiac Event)) OR (Adverse Cardiac Event)) OR (Major Adverse Cardiac Events))))	6,722,213
	#3: (systematic review OR meta-analysis)	525,958
	#4: #1 AND #2 AND #3	354
Cochrane Library	#1: ("Lymphocytes" OR "Lymphocyte" OR "Lymphoid Cells" OR "Lymphoid Cell") AND ("ratio" OR "index") OR ("systemic immune inflammation index" OR "SII" OR "systemic inflammation response index" OR "SIRI")	7,622
	#2: ("Cardiovascular Diseases" OR "MACE" OR "CVD" OR "ASCVD" OR "cardiovascular mortality" OR "cardiovascular death" OR "stroke" OR "ischemic stroke" OR "coronary" OR "heart disease" OR "CHD" OR "acute	239,331

	myocardial infarction" OR "AMI" OR "coronary artery disease" OR "CAD" OR "ischemic heart disease" OR "IHD" OR "myocardial infarction" OR "MI" OR "unstable angina" OR "UA" OR "heart failure" OR "HF" OR "Cardiac Event" OR "Cardiac Events" OR "Adverse Cardiac Event" OR "Adverse Cardiac Events" OR "Major Adverse Cardiac Events")	
	#3: ("systematic review" OR "meta-analysis")	34,541
	#4: #1 AND #2 AND #3	595
Web of Science	#1: TS=(("Lymphocytes" OR "Lymphocyte" OR "Lymphoid Cells" OR "Lymphoid Cell") AND ("ratio" OR "index")) OR TS=(("systemic immune inflammation index" OR "SII" OR "systemic inflammation response index" OR "SIRI"))	50,983
	#2: TS=(("Cardiovascular Diseases" OR "MACE" OR "CVD" OR "ASCVD" OR "cardiovascular mortality" OR "cardiovascular death" OR "stroke" OR "ischemic stroke" OR "coronary" OR "heart disease" OR "CHD" OR "acute myocardial infarction" OR "AMI" OR "coronary artery disease" OR "CAD" OR "ischemic heart disease" OR "IHD" OR "myocardial infarction" OR "MI" OR "unstable angina" OR "UA" OR "heart failure" OR "HF" OR "Cardiac Event" OR "Cardiac Events" OR "Adverse Cardiac Event" OR "Adverse Cardiac Events" OR "Major Adverse Cardiac Events"))	1,546,525
	#3: TS=("systematic review" OR "meta-analysis")	510,700
	#4: #1 AND #2 AND #3	58,351

Embase	#1: ('lymphocyte'/exp OR 'lymphocyte' OR 'lymphoid cell'/exp OR 'lymphoid cell') AND ('ratio' OR 'index') OR 'systemic immune inflammation index' OR 'sii' OR 'systemic inflammation response index' OR 'siri'	213,508
	#2: 'cardiovascular disease'/exp OR 'cardiovascular diseases' OR 'mace' OR 'cvd' OR 'ascvd' OR 'cardiovascular mortality' OR 'cardiovascular death' OR 'stroke' OR 'ischemic stroke' OR 'coronary disease'/exp OR 'heart disease'/exp OR 'chd' OR 'acute myocardial infarction'/exp OR 'ami' OR 'coronary artery disease'/exp OR 'cad' OR 'ischemic heart disease'/exp OR 'ihd' OR 'myocardial infarction'/exp OR 'mi' OR 'unstable angina'/exp OR 'ua' OR 'heart failure'/exp OR 'hf' OR 'cardiac event' OR 'cardiac events' OR 'adverse cardiac event' OR 'adverse cardiac events' OR 'major adverse cardiac events'	6,718,654
	#3:'systematic review'/exp OR 'meta-analysis'/exp	651,560
	#4: #1 AND #2 AND #3	810
The Scopus	#1: (TITLE-ABS-KEY(("Lymphocytes" OR "Lymphocyte" OR "Lymphoid Cells" OR "Lymphoid Cell") AND ("ratio" OR "index")) OR TITLE-ABS-KEY(("systemic immune inflammation index" OR "SII" OR "systemic inflammation response index" OR "SIRI")))	135,438
	#2: TITLE-ABS-KEY(("Cardiovascular Diseases" OR "MACE" OR "CVD" OR "ASCVD" OR "cardiovascular mortality" OR "cardiovascular death" OR "stroke" OR "ischemic stroke" OR "coronary" OR "heart disease" OR "CHD" OR "acute myocardial infarction" OR "AMI" OR "coronary artery disease" OR "CAD" OR "ischemic heart disease" OR "IHD" OR "myocardial infarction" OR "MI" OR "unstable angina" OR "UA" OR "heart failure" OR "HF" OR "Cardiac Event" OR "Cardiac Events" OR "Adverse Cardiac Event" OR "Adverse Cardiac Events" OR	2,845,931

	"Major Adverse Cardiac Events"))	
	#3: TITLE-ABS-KEY(("systematic review" OR "meta-analysis"))	807,223
	#4: #1 AND #2 AND #3	333
Medline	#1: ((Lymphocytes/ OR (Lymphocyte* OR "Lymphoid Cells") AND (ratio OR index)) OR ("systemic immune inflammation index" OR SII OR "systemic inflammation response index" OR SIRI))	4,830
	#2: (Cardiovascular Diseases/ OR (MACE OR CVD OR ASCVD OR "cardiovascular mortality" OR "cardiovascular death" OR stroke OR IS OR coronary OR "heart disease" OR CHD OR AMI OR "coronary artery disease" OR CAD OR "ischemic heart disease" OR IHD OR "myocardial infarction" OR MI OR "unstable angina" OR UA OR "heart failure" OR HF OR "Cardiac Event" OR "Major Adverse Cardiac Event"))	13,918
	#3: (("systematic review" OR "meta-analysis"))	9,272
	#4: #1 AND #2 AND #3	398

3. Supplementary Table S3 Characteristics of Included Systematic Reviews and Meta-Analyses

Population	Category*	Outcomes	Included SRMA	Sample size (case/total)	MA metric	Estimates [95% CI]	No. of studies (T/C/P)	Effects model	I^2; *Q* test *P*-value	Egger test *P*-value
Significant associations										
CVD	NLR	CAD	Teeranan Angkananard, 2018[1]	NA/8988	OR	1.62 [1.38, 1.91]	38/9/11	Random	53%; 0.03	0.253
CVD	NLR	ACS	Teeranan Angkananard, 2018[1]	NA/2172	OR	1.64 [1.30, 2.05]	38/9/11	Random	12.8%; 0.332	0.943
CVD	NLR	Stroke	Teeranan Angkananard, 2018[1]	NA/58867	OR	2.36 [1.44, 3.89]	38/9/11	Random	72.7%; 0.012	0.045
CVD	NLR	Composite outcomes	Teeranan Angkananard, 2018[1]	NA/5975	OR	3.86 [1.73, 8.64]	38/9/11	Random	86.7%; 0.001	0.017

MI	NLR	MACE	Nana O. Banahene, 2024[2]	NA/13156	OR	1.86 [1.53, 2.28]	37/37/0	Random	86%; NA	NA
MI	NLR	ACM	Nana O. Banahene, 2024[2]	NA/22616	OR	2.29 [1.94, 2.70]	37/37/0	Random	91%; NA	NA
Vascular Surgery	NLR	Long-term mortality	Sarah M. Jackson, 2020[3]	NA/3637	HR	1.40 [1.13, 1.74]	14/13/1	Random	60.8%; NA	NA
Vascular Surgery	NLR	Short-term mortality (30-day mortality)	Sarah M. Jackson, 2020[3]	NA/945	OR	3.08 [1.91, 4.95]	14/13/1	Random	60.8%; NA	NA
Stroke	NLR	PSI	Shokoufeh Khanzadeh, 2022[4]	876/2416	SMD	1.08 [0.78, 1.39]	15/15/0	Random	89.7%; < 0.001	0.36
Stroke	NLR	PSP	Shokoufeh Khanzadeh, 2022[4]	615/3994	SMD	0.98 [0.81, 1.14]	15/15/0	Random	65.4%; 0.003	0.28
PAD	NLR	1-year ACM	Roy B. Kurniawan,	NA	RR	2.54 [1.64, 3.95]	8/8/0	Random	79%; < 0.01	NA

			2024[5]							
PAD	NLR	5-year ACM	Roy B. Kurniawan, 2024[5]	NA	RR	1.45 [1.23, 1.72]	8/8/0	Random	0%; < 0.76	NA
PAD	NLR	1-year MALE	Roy B. Kurniawan, 2024[5]	NA	RR	3.91 [1.85, 8.25]	6/6/0	Random	87%; < 0.01	NA
Cardiac Surgery	Preoperative NLR	POAF	Zhengyang Liu, 2020[6]	NA/9262	OR	1.42 [1.16, 1.72]	12/12/0	Random	99.05%; < 0.01	0.4017
ACS undergoing PCI	NLR	Severity of coronary involvement	Farzad Shahsanaei, 2024[7]	NA/20846	OR	1.175 [1.021, 1.353]	14/14/0	Random	62.72%; 0.002	0.035 to 0.045
ACS undergoing PCI	NLR	Long-term mortality	Farzad Shahsanaei, 2024[7]	NA/20846	OR	3.424 [2.325, 5.025]	14/14/0	Random	79.53%; 0.001	0.035 to 0.045
ACS undergoing	NLR	Long-term MACE	Farzad Shahsanaei,	NA/20846	OR	2.604 [1.736, 3.906]	14/14/0	Random	90.61%; 0.001	0.035 to 0.045

PCI			2024[7]							
aSAH	NLR	Poor functional outcome	Min Shi, 2021[8]	NA/4018	OR	1.32 [1.11, 1.57]	10/10/0	Random	87%; < 0.00001	NA
aSAH	NLR	DCI	Min Shi, 2021[8]	NA/2466	OR	1.72 [1.22, 2.41]	10/10/0	Random	82%; 0.0002	NA
Angiography or cardiac revascularization	NLR	ACM	Xiaoding Wang, 2014[9]	NA/4396	OR	2.33 [1.88, 2.88]	8/8/0	Fixed	31%; 0.18	NA
Angiography or cardiac revascularization	NLR	Cardiovascular events	Xiaoding Wang, 2014[9]	NA/8789	OR	1.89 [1.42, 2.52]	5/5/0	Random	55%; 0.06	NA
Aortic diseases	NLR	Mortality (High NLR group vs. low NLR group)	Yan Xu, 2020[10]	370/2963	RR	2.63 [1.79, 3.86]	7/7/0	Random	67%; 0.001	NA

AIS	NLR	HT	Ruirui Zhang, 2019[11]	NA/3726	OR	1.53 [1.21, 1.92]	7/7/0	Random	86%; < 0.00001	NA
STEMI	NLR	Overall mortality	Francesco Dentali, 2018[12]	NA/16000	OR	4.60 [2.84, 7.45]	23/23/0	Random	74%; < 0.00001	NA
NSTEMI	NLR	Overall mortality	Francesco Dentali, 2018[12]	NA/16000	OR	6.41 [2.65, 15.50]	23/23/0	Random	70%; NA	NA
STEMI	NLR	MACE	Francesco Dentali, 2018[12]	NA/16000	OR	3.71 [2.67, 5.17]	23/23/0	Random	55%; NA	NA
AIS	NLR	Mortality	Li, 2021[13]	NA/11124	OR	1.12 [1.07, 1.16]	41/41/0	Random	76%; < 0.00001	< 0.05
AIS	NLR	Poor Outcome	Li, 2021[13]	NA/5679	OR	1.29 [1.16, 1.44]	41/41/0	Random	82%; < 0.00001	< 0.05
AIS	NLR	sICH/HT	Li, 2021[13]	NA/4539	OR	1.15 [1.08, 1.32]	41/41/0	Random	69%; < 0.0001	< 0.05
AHS	NLR	Mortality	Li, 2021[13]	NA/2957	OR	1.23 [1.09, 1.39]	41/41/0	Random	92%; < 0.00001	NA
AHS	NLR	Poor Outcome	Li, 2021[13]	NA/2733	OR	1.11 [1.03, 1.20]	41/41/0	Random	93%; < 0.00001	NA

AF	NLR	Stroke risk stratification	Lu M, 2022[14]	NA/941	WMD	0.72 [0.43, 1.01]	6/0/6	Random	69.7%; 0.006	NA
AF	NLR	With or without stroke	Lu M, 2022[14]	NA/1268	WMD	1.96 [1.38, 2.53]	5/0/5	Random	54.7%; 0.066	0.334
AF	NLR	Stroke incidence	Lu M, 2022[14]	NA/32912	RR	1.4 [1.24, 1.58]	1/1/0	Fixed	0%; NA	NA
STEMI	NLR	ACM	Rodry Mikhael, 2020[15]	NA/3250	OR	2.74 [1.99, 3.77]	12/12/0	Fixed	36%; NA	NA
STEMI	NLR	Cardiac-related mortality	Rodry Mikhael, 2020[15]	NA/4333	OR	3.20 [2.47, 4.14]	12/12/0	Fixed	0%; NA	NA
ACS	NLR	Mortality	Michał Pruc, 2024[16]	1425/10768	SMD	-2.55 [-3.90, -1.19]	12/12/0	Random	100%; < 0.00001	NA
STEMI	NLR	Mortality	Michał Pruc, 2024[16]	573/4937	SMD	-1.94 [-2.82, -1.07]	10/10/0	Random	99%; < 0.00001	NA
AIS	NLR	Functional	Si-Ying Song,	NA/5858	HR	1.76 [1.40, 2.21]	17/17/0	Random	89%; 0.000	NA

		outcome	2019[17]							
AIS	NLR	Mortality	Si-Ying Song, 2019[17]	NA/7517	HR	1.08 [1.04, 1.13]	20/20/0	Random	69.8%; 0.000	NA
AHS	NLR	Mortality	Si-Ying Song, 2019[17]	NA/7517	HR	1.09 [1.03, 1.16]	20/20/0	Random	88.1%; 0.000	NA
STEMI undergoing PCI	NLR	In-hospital ACM	Hassan Ul Hussain, 2024[18]	NA	RR	3.52 [2.93, 4.24]	18/18/0	Random	7%; 0.37	NA
STEMI undergoing PCI	NLR	long-term MACE	Hassan Ul Hussain, 2024[18]	NA	RR	2.92 [2.16, 3.94]	6/6/0	Random	41%; 0.15	NA
Acute stroke	NLR	Poor functional outcome at 3 months	Jinzhao Wan, 2020[19]	NA/4443	OR	1.26 [1.15, 1.38]	13/13/0	Random	86.9%; < 0.001	< 0.001
Acute stroke	NLR	Mortality at 3 months	Jinzhao Wan, 2020[19]	NA/4443	OR	1.63 [1.15, 2.31]	13/13/0	Random	90.6%; < 0.001	< 0.001

AIS treated with IVT	NLR	HT	Chengbing Wang, 2021[20]	NA/2041	OR	1.33 [1.14, 1.56]	6/6/0	Random	71.8%; < 0.001	0.019
AIS treated with IVT	NLR	Poor functional outcome at 3 months	Chengbing Wang, 2021[20]	NA/3641	OR	1.64 [1.38, 1.94]	10/10/0	Random	86.3%; < 0.001	0.001
AIS treated with RT	Admission NLR	PFO	Bing Wu, 2023[21]	NA/8474	OR	1.13 [1.09, 1.17]	26/26/0	Random	75%; NA	< 0.001
AIS treated with RT	Post-treat ment NLR	PFO	Bing Wu, 2023[21]	NA/3686	OR	1.25 [1.16, 1.35]	14/14/0	Random	86.6%; NA	< 0.001
AIS treated with RT	Admission NLR	sICH	Bing Wu, 2023[21]	NA/6977	OR	1.11 [1.06, 1.16]	16/16/0	Random	71.6%; NA	< 0.001
AIS treated with RT	Post-treat ment NLR	sICH	Bing Wu, 2023[21]	NA/1568	OR	1.14 [1.01, 1.29]	4/4/0	Random	80.8%; NA	0.005
AIS treated with RT	Admission NLR	3-month mortality	Bing Wu, 2023[21]	NA/6473	OR	1.13 [1.07, 1.20]	14/14/0	Random	79%; NA	< 0.001

AIS treated with RT	Post-treatment NLR	3-month mortality	Bing Wu, 2023[21]	NA/2274	OR	1.28 [1.09, 1.50]	6/6/0	Random	91.7%; NA	NA
ACS	SII	Short-term ACM	Shengpeng Wang, 2024[22]	NA/16596	HR	2.60 [1.29, 5.25]	11/11/0	Random	84.4%; < 0.001	NA
ACS	SII	Long-term ACM	Shengpeng Wang, 2024[22]	NA/16596	HR	2.40 [1.25, 4.59]	11/11/0	Random	89.9%; < 0.001	NA
ACS	SII	Short-term MACEs	Shengpeng Wang, 2024[22]	NA/16596	HR	1.61 [1.28, 2.03]	11/11/0	Random	0%; 0.498	NA
ACS	SII	Long-term MACEs	Shengpeng Wang, 2024[22]	NA/16596	HR	2.43 [1.74, 3.40]	11/11/0	Random	73.6%; 0.001	0.027
Stroke	SII	Poor outcome	Yong-Wei Huang, 2022[23]	NA/18609	OR	1.06 [1.02, 1.09]	19/19/0	Random	93%; < 0.00001	NA
Stroke	SII	Mortality	Yong-Wei Huang, 2022[23]	NA/18609	OR	2.16 [1.75, 2.67]	19/19/0	Random	49%; 0.16	NA
Stroke	SII	HT	Yong-Wei Huang, 2022[23]	NA/18609	OR	2.09 [1.61, 2.71]	19/19/0	Random	42%; 0.16	NA

Different levels of SII	SII	ACM	Wei Li, 2024[24]	NA/427819	HR	1.45 [1.36, 1.54]	33/33/0	Random	79.8%; 0.000	< 0.01
Different levels of SII	SII	CVD mortality	Wei Li, 2024[24]	NA/378420	HR	1.44 [1.29, 1.60]	11/11/0	Random	64.9%; 0.001	0.108
Different levels of SII	SII	CVD risk	Zhen Ye, 2022[25]	NA/151105	HR	1.39 [1.20, 1.61]	8/8/0	Random	90.8%; < 0.001	0.781
Patients undergoing PCI	SII	MACEs	Chunyu Zhang, 2024[26]	NA/11117	RR	2.08 [1.87, 2.32]	8/8/0	Fixed	42%; < 0.00001	NA
Patients undergoing PCI	SII	ACM	Chunyu Zhang, 2024[26]	NA/11117	RR	4.71 [2.75, 8.08]	3/3/0	Random	76%; < 0.00001	NA
Patients undergoing PCI	SII	Non-fatal MI	Chunyu Zhang, 2024[26]	NA/11117	RR	1.84 [1.36, 2.48]	4/4/0	Random	51%; < 0.0001	NA

Patients undergoing PCI	SII	HF	Chunyu Zhang, 2024[26]	NA/11117	RR	1.61 [1.39, 1.86]	3/3/0	Fixed	21%; < 0.00001	NA
CAD	SII	MACE	Zehao Zhao, 2024[27]	NA/9510	HR	2.36 [1.67, 3.33]	6/6/0	Random	80%; < 0.01	0.0369
CAD	SII	ACM	Zehao Zhao, 2024[27]	NA/3271	HR	3.07 [2.00, 4.72]	4/4/0	Random	51%; 0.09	NA
CAD	SII	Cardiovascular mortality	Zehao Zhao, 2024[27]	NA/2012	HR	2.70 [1.40, 5.18]	3/3/0	Random	83%; < 0.01	NA
CAD	SII	MI	Zehao Zhao, 2024[27]	NA/2908	HR	1.85 [1.24, 2.79]	4/4/0	Random	68%; 0.02	NA
CAD	SII	Stroke	Zehao Zhao, 2024[27]	NA/1875	HR	2.25 [1.16, 4.35]	3/3/0	Random	67%; 0.03	NA
STEMI patients after pPCI	PLR	MACE (In-hospital)	Guoxia Dong, 2021[28]	NA	RR	1.76 [1.39, 2.22]	6/6/0	Random	49%; < 0.001	NA

STEMI patients after pPCI	PLR	ACM (In-hospital)	Guoxia Dong, 2021[28]	NA	RR	1.91 [1.18, 3.09]	2/2/0	Random	0%; 0.009	NA
STEMI patients after pPCI	PLR	cardiac mortality (In-hospital)	Guoxia Dong, 2021[28]	NA	RR	2.14 [1.52, 3.01]	7/7/0	Random	24%; < 0.001	NA
STEMI patients after pPCI	PLR	no reflow after PCI (In-hospital)	Guoxia Dong, 2021[28]	NA	RR	2.22 [1.70, 2.90]	6/6/0	Random	59%; < 0.001	NA
STEMI patients after pPCI	PLR	MACE (Long-term)	Guoxia Dong, 2021[28]	NA	RR	1.60 [1.25, 2.03]	4/4/0	Random	57%; < 0.001	NA
STEMI patients after pPCI	PLR	ACM (Long-term)	Guoxia Dong, 2021[28]	NA	RR	2.36 [1.53, 3.66]	5/5/0	Random	78%; < 0.001	NA
AIS treated with RT	Admission PLR	90-day Good Functional	Divyansh Sharma, 2022[29]	NA/996	SMD	-0.32 [-0.58, -0.05]	12/12/0	Random	75%; 0.007	NA

		Outcomes								
AIS treated with RT	Delayed PLR	90-day Good Functional Outcomes	Divyansh Sharma, 2022[29]	NA/1297	SMD	-0.43 [-0.54, -0.32]	12/12/0	Random	0%; 0.68	NA
AIS treated with RT	PLR	ENI	Divyansh Sharma, 2022[29]	NA/1475	SMD	-0.18 [-0.29, -0.08]	12/12/0	Random	0%; 0.656	NA
HF	PLR	Follow-up mortality	Mehrbod Vakhshoori, 2024[30]	NA/8159	MD	162.55 [149.35, 175.75]	16/16/0	Random	98.04%; < 0.001	0.002
HF	PLR	In-hospital mortality	Mehrbod Vakhshoori, 2024[30]	NA/13924	MD	192.83 [150.06, 235.61]	5/5/0	Random	98.19%; < 0.001	0.0335
AIS	SIRI	Poor functional outcome at 3 months	Ying Han and Nan Lin, 2024[31]	1389/4062	OR	1.57 [1.39, 1.78]	14/14/0	Random	0%; < 0.001	0.43

AIS	SIRI	Poor functional outcome	Yong-Wei Huang, 2022[32]	1099/1989	OR	3.01 [2.00, 4.54]	11/11/0	Random	74%; < 0.0001	NA
AIS	SIRI	SAP	Yong-Wei Huang, 2022[32]	82/540	OR	2.91 [2.21, 3.75]	2/2/0	Random	0%; 0.66	NA
AIS	SIRI	END	Yong-Wei Huang, 2022[32]	68/323	OR	3.79 [2.14, 6.74]	2/2/0	Random	85%; 0.01	NA
Non-significant associations										
Cardiac Surgery	Postoperative NLR	POAF	Zhengyang Liu, 2020[6]	NA/922	SMD	1.60 [-0.56, 3.77]	12/12/0	Random	51.20%; NA	0.4017
AIS treated with IVT	NLR	3-month mortality	Chengbing Wang, 2021[20]	NA/1459	OR	1.14 [0.97, 1.35]	4/4/0	Random	81%; < 0.001	NA
NSTEMI	NLR	Mortality	Michał Pruc, 2024[16]	234/543	SMD	-0.63 [-2.54, 1.28]	3/3/0	Random	98%; < 0.00001	NA
PAD	NLR	4-year MALE	Roy B. Kurniawan,	NA	RR	1.29 [0.96, 1.73]	6/6/0	Random	92%; < 0.01	NA

			2024[5]							
AAD	NLR	NLR values in the hospital death team compared to the survival team	Zekun Li, 2023[33]	NA/553	OR	1.89 [0.87, 2.98]	8/0/8	Fixed	21%; > 0.10	NA
AAD	NLR	In-hospital mortality (High vs. low)	Zekun Li, 2023[33]	NA/808	OR	1.20 [0.55, 2.14]	8/0/8	Fixed	NA	NA
Atrial Fibrillation Ablation	Preablatio n NLR	AF recurrence	Sai Prasanna Lekkala, 2023[34]	NA/726	OR	1.45 [0.87, 2.43]	3/3/0	Random	95.1%; < 0.01	NA
Atrial Fibrillation Ablation	Postablatio n NLR	AF recurrence	Sai Prasanna Lekkala, 2023[34]	NA/878	OR	1.28 [0.93, 1.76]	2/2/0	Random	95.32%; < 0.01	NA

AHS	NLR	Functional outcome	Si-Ying Song, 2019[17]	NA/5858	HR	1.02 [0.79, 1.08]	17/17/0	Random	77.8%; 0.004	NA
STEMI undergoing PCI	NLR	Long-term any revascularization	Hassan Ul Hussain, 2024[18]	NA	RR	1.17 [1.00, 1.37]	4/4/0	Random	19%; 0.29	NA
HF	PLR	Long-term mortality risk	Mehrbod Vakhshoori, 2024[30]	NA/13924	HR	1.02 [0.99, 1.05]	NA	Fixed	NA	NA
Patients undergoing cardiothoracic surgery	PLR	POAF incidence after all surgeries	Biling Ye, 2024[35]	NA/3195	OR	1.01 [1.00, 1.01]	6/1/5	Random	43.4%; 0.116	0.652
Patients undergoing cardiothoracic surgery	PLR	POAF incidence specifically after CABG	Biling Ye, 2024[35]	NA/3195	OR	1.01 [1.00, 1.02]	6/1/5	Random	0%; 0.894	0.652
Stroke	SII	Recanalizati	Yong-Wei	NA/18609	OR	1.50 [0.86, 2.62]	19/19/0	Random	74%; 0.05	NA

		on	Huang, 2022[23]							
Patients undergoing PCI	SII	Non-fatal stroke	Chunyu Zhang, 2024[26]	NA/11117	RR	2.34 [0.64, 8.51]	3/3/0	Random	93%; 0.20	NA
Patients undergoing PCI	SII	Repeat revasculariz ation	Chunyu Zhang, 2024[26]	NA/11117	RR	1.19 [0.78, 1.83]	4/4/0	Random	89%; 0.41	NA
CAD	SII	Ischemia-dri ven revasculariz ation	Zehao Zhao, 2024[27]	NA/2100	HR	1.66 [0.98, 2.83]	3/3/0	Random	87%; 0.41	NA
AIS treated with RT	PLR	Radiological Bleed	Divyansh Sharma, 2022[29]	NA/505	SMD	0.27 [-0.15, 0.70]	12/12/0	Random	71.6%; 0.03	NA
AIS	SIRI	ACM	Yong-Wei Huang, 2022[32]	140/526	OR	1.90 [0.84, 4.24]	4/4/0	Random	81%; 0.001	NA

Note: CI = confidence interval; OR = odds ratio; HR = hazard ratio; RR = risk ratio; SMD = standardized mean difference; WMD = weighted mean difference; MD = mean difference; I² = heterogeneity index; MA metric = meta-analysis metric; NA = not available or not applicable; T = total number of studies; C = cohort studies; P = population-based case-control and/or cross-sectional studies; CVD = cardiovascular disease; CAD = coronary artery disease; ACS = acute coronary syndrome; MI = myocardial infarction; STEMI = ST-elevation myocardial

infarction; NSTEMI = non-ST-elevation myocardial infarction; PAD = peripheral artery disease; AAD = acute aortic dissection; PCI = percutaneous coronary intervention; pPCI = primary percutaneous coronary intervention; CABG = coronary artery bypass grafting; HF = heart failure; MACE = major adverse cardiovascular events; ACM = all-cause mortality; MALE = major adverse limb events; AIS = acute ischemic stroke; AHS = acute hemorrhagic stroke; aSAH = aneurysmal subarachnoid hemorrhage; HT = hemorrhagic transformation; sICH = symptomatic intracerebral hemorrhage; END = early neurological deterioration; SAP = stroke-associated pneumonia; PSI = pneumonia severity index; PSP = post-stroke pneumonia; ENI = early neurological improvement; PFO = patent foramen ovale; AF = atrial fibrillation; POAF = postoperative atrial fibrillation; IVT = intravenous thrombolysis; RT = reperfusion therapy; IVIG = intravenous immunoglobulin; DCI = delayed cerebral ischemia; Recanalization = restoration of blood flow; NLR = neutrophil-to-lymphocyte ratio; PLR = platelet-to-lymphocyte ratio; SII = systemic immune-inflammation index; SIRI = systemic inflammatory response index; *Category = Immune-inflammatory indices.

4. Supplementary Table S4 Assessments of AMSTAR-2 Scores

Study first and second author	Item 1	Item 2	Item 3	Item 4	Item 5	Item 6	Item 7	Item 8	Item 9	Item 10	Item 11	Item 12	Item 13	Item 14	Item 15	Item 16	Overall Rating
Teeranan Angkananard, Thunyarat Anothaisintawee	Yes	Yes	Yes	Yes	Yes	Yes	Yes	Yes	Yes	Yes	Yes	Yes	Yes	Yes	Yes	Yes	High
Nana O. Banahene Tanya Sinha	Yes	No	Yes	Yes	Yes	Yes	Yes	Yes	Yes	Yes	Yes	Yes	Yes	Yes	Yes	Yes	Moderate
Sarah M. Jackson Luke A. Perry	Yes	No	Yes	Yes	Yes	Yes	Yes	Yes	Yes	Yes	Yes	Yes	Yes	Yes	Yes	Yes	Moderate
Shokoufeh Khanzadeh Brandon Lucke-Wold	Yes	No	Yes	Yes	Yes	Yes	Yes	Yes	Yes	Yes	Yes	Yes	Yes	Yes	Yes	Yes	High
Roy B Kurniawan Paulus P Siahaan	Yes	Yes	Yes	Yes	Yes	Yes	Yes	Yes	Yes	Yes	Yes	Yes	Yes	Yes	Yes	Yes	High
Zekun Li Danchun Lian	Yes	No	Yes	Yes	Yes	Yes	Yes	Yes	Yes	Yes	Yes	Yes	Yes	No	Yes	No	Moderate
Zhengyang Liu Jacqueline Nguyen Khuong	Yes	Yes	Yes	Yes	Yes	Yes	Yes	Yes	Yes	Yes	Yes	Yes	Yes	Yes	Yes	Yes	High
Farzad Shahsanaei Shahin Abbaszadeh	Yes	No	Yes	Yes	Yes	Yes	Yes	Yes	Yes	Yes	Yes	Yes	Yes	Yes	Yes	Yes	Moderate

Min Shi<br>Chao Yang	Yes	No	Yes	Yes	Yes	Yes	Yes	Yes	Yes	Yes	Yes	Yes	Yes	Yes	Yes	Yes	Moderate
Xiaoding Wang<br>Guangyu Zhang	Yes	No	Yes	No	Yes	Yes	Yes	Yes	Yes	Yes	Yes	Yes	Yes	No	Yes	No	Low
Yan Xu<br>Haiyang Fang	Yes	No	Yes	Yes	Yes	Yes	Yes	Yes	Yes	Yes	Yes	Yes	Yes	Yes	Yes	Yes	Moderate
Ruirui Zhang<br>Xiaodong Wu	Yes	No	No	Yes	Yes	Yes	Yes	Yes	Yes	Yes	Yes	Yes	Yes	No	Yes	No	Moderate
Francesco Dentali<br>Olga Nigro	Yes	No	Yes	Yes	Yes	Yes	Yes	Yes	Yes	Yes	Yes	Yes	Yes	No	Yes	No	Moderate
Sai Prasanna Lekkala<br>Sai Priyanka Mellacheruvu	Yes	No	Yes	Yes	Yes	Yes	Yes	Yes	Yes	Yes	Yes	Yes	No	No	Yes	No	Moderate
Wenxia Li<br>Miaomiao Hou	Yes	Yes	Yes	Yes	Yes	Yes	Yes	Yes	Yes	Yes	Yes	Yes	Yes	Yes	Yes	Yes	High
Ming Lu<br>Yeying Zhang	Yes	No	Yes	Yes	Yes	Yes	Yes	Yes	Yes	Yes	Yes	Yes	Yes	Yes	Yes	Yes	Moderate
Rodry Mikhael<br>Evan Hindoro	Yes	No	Yes	Yes	Yes	Yes	Yes	Yes	Yes	Yes	Yes	No	Yes	No	Yes	No	Low
Michał Pruc<br>Jacek Kubica	Yes	Yes	Yes	Yes	Yes	Yes	Yes	Yes	Yes	Yes	Yes	Yes	Yes	No	Yes	No	High
Si-Ying Song<br>Xiao-Xi Zhao	Yes	Yes	Yes	Yes	Yes	Yes	Yes	Yes	Yes	Yes	Yes	Yes	Yes	No	Yes	No	High
Hassan Ul Hussain<br>Kanwal	Yes	Yes	Yes	Yes	Yes	Yes	Yes	Yes	Yes	Yes	Yes	Yes	Yes	No	Yes	No	High

Ashok Kumar																	
Jinzhao Wan Xiaoxiong Wang	Yes	No	Yes	Yes	Yes	Yes	Yes	Yes	Yes	Yes	Yes	Yes	Yes	Yes	Yes	Yes	Moderate
Chengbing Wang Qian Zhang	Yes	No	Yes	Yes	Yes	Yes	Yes	Yes	Yes	Yes	Yes	Yes	Yes	Yes	Yes	Yes	Moderate
Bing Wu Fang Liu	Yes	Yes	Yes	Yes	Yes	Yes	Yes	Yes	Yes	Yes	Yes	Yes	Yes	Yes	Yes	Yes	High
Shengpeng Wang Guannan Zhang	Yes	No	Yes	Yes	Yes	Yes	Yes	Yes	Yes	Yes	Yes	Yes	Yes	Yes	Yes	Yes	Moderate
Yong-Wei Huang Xiao-Shuang Yin	Yes	Yes	Yes	Yes	Yes	Yes	Yes	Yes	Yes	Yes	Yes	Yes	Yes	Yes	Yes	Yes	High
Wei Li Xiaoning Wang	Yes	Yes	Yes	Yes	Yes	Yes	Yes	Yes	Yes	Yes	Yes	Yes	Yes	Yes	Yes	Yes	High
Zhen Ye Tingyi Hu	Yes	No	Yes	Yes	Yes	Yes	Yes	Yes	Yes	Yes	Yes	Yes	Yes	Yes	Yes	Yes	Moderate
Chunyu Zhang Minghao Li	Yes	Yes	Yes	Yes	Yes	Yes	Yes	Yes	Yes	Yes	Yes	Yes	Yes	Yes	Yes	Yes	High
Zehao Zhao Xiaoming Zhang	Yes	Yes	Yes	Yes	Yes	Yes	Yes	Yes	Yes	Yes	Yes	Yes	Yes	Yes	Yes	Yes	High
Guoxia Dong Aiqin Huang	Yes	No	Yes	Yes	Yes	Yes	Yes	Yes	Yes	Yes	Yes	Yes	Yes	Yes	Yes	Yes	Moderate
Ying Han Nan Lin	Yes	No	Yes	Yes	Yes	Yes	Yes	Yes	Yes	Yes	Yes	Yes	Yes	Yes	Yes	Yes	Moderate

Yong-Wei Huang<br>Ye Zhang	Yes	Yes	Yes	Yes	Yes	Yes	Yes	Yes	Yes	Yes	Yes	Yes	Yes	Yes	Yes	Yes	High
Divyansh Sharma<br>Sonu M. M. Bhaskar	Yes	No	Yes	Yes	Yes	Yes	Yes	Yes	Yes	Yes	Yes	Yes	Yes	Yes	Yes	Yes	Moderate
Mehrbod Vakhshoori<br>Niloofar Bondariyan	Yes	Yes	Yes	Yes	Yes	Yes	Yes	Yes	Yes	Yes	Yes	Yes	Yes	Yes	Yes	Yes	High
Biling Ye<br>Junping Gan	Yes	No	Yes	Yes	Yes	Yes	Yes	Yes	Yes	Yes	Yes	Yes	Yes	Yes	Yes	Yes	Moderate

Note: Item 1: Inclusion of PICO elements? Item 2: Review methods established before conduct of review? Item 3: Explanation for selection of study designs to be included in review? Item 4: Use of a comprehensive search strategy? Item 5: Selection of studies in duplicate? Item 6: Data extraction in duplicate? Item 7: Provision of list of excluded studies with justification for exclusion? Item 8: Description of included studies in adequate detail? Item 9: Satisfactory technique for risk of bias? Item 10: Sources of funding for included studies reported? Item 11: Proper methods for meta-analysis? Item 12: Potential risk of bias in included studies discussed? Item 13: Risk of bias accounted for in interpreting results? Item 14: Heterogeneity discussed? Item 15: If meta-analysis conducted was publication bias discussed? Item 16: Disclosure of funding or conflict of interest? NA: not applicable (meta-analysis not performed to score this category).

5. Supplementary Table S5 GRADE Classification of Quality of Evidence

Population	Category*	Comparison	Outcomes	Included MA	No. of studies (T/C/P)	Risk of bias	Inconsistency	Indirectness	Imprecision	Publication bias	Plausible confounding	Magnitude of effect	Dose-response gradient	Quality
Significant associations														
CVD	NLR	High vs. low	CAD	Teerananangkananard, 2018	38/9/11	No serious risk	Serious inconsistency	No serious indirectness	Serious imprecision	Undetected	Would not reduce effect	No	No	Very low
CVD	NLR	High vs. low	ACS	Teerananangkananard, 2018	38/9/11	No serious risk	No serious inconsistency	No serious indirectness	Serious imprecision	Undetected	Would not reduce effect	No	No	Very low
CVD	NLR	High vs. low	Stroke	Teerananangkananard,	38/9/11	No serious	Serious inconsi	No serious indirec	Serious impreci	Strongly suspe	Would not reduce	Yes	No	Very low

				2018		risk	stency	tness	sion	cted	effect			
CVD	NLR	High vs. low	Composite outcomes	Teeranan Angkananard, 2018	38/9/11	No serious risk	Serious inconsistency	No serious indirectness	Serious imprecision	Strongly suspected	Would not reduce effect	Yes	No	Very low
MI	NLR	Elevated vs. without elevated	MACE	Nana O. Banahene, 2024.	37/37/0	No serious risk	Serious inconsistency	No serious indirectness	No serious imprecision	NA	Would not reduce effect	No	No	Low
MI	NLR	Elevated vs. without elevated	ACM	Nana O. Banahene, 2024.	37/37/0	No serious risk	Serious inconsistency	No serious indirectness	No serious imprecision	NA	Would not reduce effect	Yes	No	Moderate
Vascular Surgery	NLR	Elevated preoperative vs. without elevated	Long-term mortality	Sarah M. Jackson, 2020	14/13/1	No serious risk	Serious inconsistency	No serious indirectness	Serious imprecision	NA	Would not reduce effect	No	No	Very low

Vascular Surgery	NLR	Elevated preoperative vs. without elevated	Short-term mortality (30-day mortality)	Sarah M. Jackson, 2020	14/13/1	No serious risk	Serious inconsistency	No serious indirectness	Serious imprecision	NA	Would not reduce effect	Yes	No	Low
Stroke	NLR	NLR level between PSI and NPSI	PSI	Shokoufeh Khanzadeh, 2022	15/15/0	No serious risk	Serious inconsistency	No serious indirectness	No serious imprecision	Undetected	Would not reduce effect	No	No	Low
Stroke	NLR	NLR level between PSP and NPSP	PSP	Shokoufeh Khanzadeh, 2022	15/15/0	No serious risk	Serious inconsistency	No serious indirectness	No serious imprecision	Undetected	Would not reduce effect	No	No	Low
PAD	NLR	High vs. low	1-year ACM	Roy B. Kurniawan, 2024	8/8/0	No serious risk	Serious inconsistency	No serious indirectness	No serious imprecision	NA	Would not reduce effect	Yes	No	Moderate
PAD	NLR	High vs. low	5-year ACM	Roy B. Kurniaw	8/8/0	No serious	No serious inconsi	No serious indirec	No serious impreci	NA	Would not reduce	No	No	Low

				an, 2024		risk	stency	tness	sion		effect			
PAD	NLR	High vs. low	1-year MALE	Roy B. Kurniawan, 2024	6/6/0	No serious risk	Serious inconsistency	No serious indirectness	No serious imprecision	NA	Would not reduce effect	Yes	No	Moderate
Cardiac Surgery	Preoperative NLR	Preoperative elevated vs. without elevated	POAF	Zhengyang Liu, 2020	12/12/0	No serious risk	Serious inconsistency	No serious indirectness	No serious imprecision	Undetected	Would not reduce effect	No	No	Low
ACS undergoing PCI	NLR	High vs. low	Severity of coronary involvement	Farzad Shahsanaei, 2024	14/14/0	No serious risk	Serious inconsistency	No serious indirectness	Serious imprecision	Strongly suspected	Would not reduce effect	No	No	Very low
ACS undergoing PCI	NLR	High vs. low	Long-term mortality	Farzad Shahsanaei, 2024	14/14/0	No serious risk	Serious inconsistency	No serious indirectness	Serious imprecision	Strongly suspected	Would not reduce effect	Yes	No	Very low
ACS undergoing	NLR	High vs.	Long-term	Farzad Shahsan	14/14/0	No serious	Serious inconsi	No serious	Serious impreci	Strongly	Would not	Yes	No	Very

PCI		low	MACE	aei, 2024		risk	stency	indirectness	sion	suspected	reduce effect			low
aSAH	NLR	High vs. low	Poor functional outcome	Min Shi, 2021	10/10/0	No serious risk	Serious inconsistency	No serious indirectness	Serious imprecision	NA	Would not reduce effect	No	No	Very low
aSAH	NLR	High vs. low	DCI	Min Shi, 2021	10/10/0	No serious risk	Serious inconsistency	No serious indirectness	Serious imprecision	NA	Would not reduce effect	No	No	Very low
Angiography or cardiac revascularization	NLR	Highest vs. lowest	ACM	Xiaoding Wang, 2014	8/8/0	No serious risk	No serious inconsistency	No serious indirectness	No serious imprecision	NA	Would not reduce effect	Yes	No	Moderate
Angiography or cardiac revasculari	NLR	Highest vs. lowest	Cardiovascular events	Xiaoding Wang, 2014	5/5/0	No serious risk	Serious inconsistency	No serious indirectness	No serious imprecision	NA	Would not reduce effect	No	No	Low

zation													
Aortic diseases	NLR	High vs. low	Mortality (High NLR group vs. low NLR group)	Yan Xu, 2020	7/7/0	No serious risk	Serious inconsistency	No serious indirectness	No serious imprecision	NA	Would not reduce effect	Yes	No	Moderate
AIS	NLR	NLR above the cutoff value for HT in patients with AIS	HT	Ruirui Zhang, 2019	7/7/0	No serious risk	Serious inconsistency	No serious indirectness	Serious imprecision	NA	Would not reduce effect	No	No	Very low
STEMI	NLR	High vs. low	Overall mortality	Francesco Dentali, 2018	23/23/0	No serious risk	Serious inconsistency	No serious indirectness	No serious imprecision	NA	Would not reduce effect	Yes	No	Moderate
NSTEMI	NLR	High vs. low	Overall mortality	Francesco Dentali,	23/23/0	No serious	Serious inconsi	No serious indirec	No serious impreci	NA	Would not reduce	Yes	No	Moderate

				2018		risk	stency	tness	sion		effect			
STEMI	NLR	High vs. low	MACE	Francesco Dentali, 2018	23/23/0	No serious risk	Serious inconsistency	No serious indirectness	No serious imprecision	NA	Would not reduce effect	Yes	No	Moderate
AIS	NLR	High vs. low	Mortality	Li, 2021	41/41/0	No serious risk	Serious inconsistency	No serious indirectness	No serious imprecision	Strongly suspected	Would not reduce effect	No	No	Very low
AIS	NLR	High vs. low	Poor Outcome	Li, 2021	41/41/0	No serious risk	Serious inconsistency	No serious indirectness	No serious imprecision	Strongly suspected	Would not reduce effect	No	No	Very low
AIS	NLR	High vs. low	sICH/HT	Li, 2021	41/41/0	No serious risk	Serious inconsistency	No serious indirectness	No serious imprecision	Strongly suspected	Would not reduce effect	No	No	Very low
AHS	NLR	High vs.	Mortality	Li, 2021	41/41/0	No serious	Serious inconsi	No serious	Serious impreci	NA	Would not	No	No	Very

		low				risk	stency	indirectness	sion		reduce effect			low
AHS	NLR	High vs. low	Poor Outcome	Li, 2021	41/41/0	No serious risk	Serious inconsistency	No serious indirectness	Serious imprecision	NA	Would not reduce effect	No	No	Very low
AF	NLR	NLR levels	Stroke risk stratification	Lu M, 2022	6/0/6	No serious risk	Serious inconsistency	No serious indirectness	No serious imprecision	NA	Would not reduce effect	No	No	Low
AF	NLR	NLR levels	With or without stroke	Lu M, 2022	5/0/5	No serious risk	Serious inconsistency	No serious indirectness	No serious imprecision	Undetected	Would not reduce effect	No	No	Low
AF	NLR	NLR levels	Stroke incidence	Lu M, 2022	1/1/0	No serious risk	No serious inconsistency	No serious indirectness	No serious imprecision	NA	Would not reduce effect	No	No	Low

STEMI	NLR	High vs. low	ACM	Rodry Mikhael, 2020	12/12/0	No serious risk	No serious inconsistency	No serious indirectness	No serious imprecision	NA	Would not reduce effect	Yes	No	Moderate
STEMI	NLR	High vs. low	Cardiac-related mortality	Rodry Mikhael, 2020	12/12/0	No serious risk	No serious inconsistency	No serious indirectness	No serious imprecision	NA	Would not reduce effect	Yes	No	Moderate
ACS	NLR	High vs. low	Mortality	Michał Pruc, 2024	12/12/0	Serious risk	Serious inconsistency	No serious indirectness	No serious imprecision	Strongly suspected	Would not reduce effect	No	No	Very low
STEMI	NLR	High vs. low	Mortality	Michał Pruc, 2024	10/10/0	Serious risk	Serious inconsistency	No serious indirectness	Serious imprecision	Strongly suspected	Would not reduce effect	No	No	Very low
AIS	NLR	High vs. low	Functional outcome	Si-Ying Song, 2019	17/17/0	No serious risk	Serious inconsistency	No serious indirec	No serious impreci	NA	Would not reduce	No	No	Low

								tness	sion		effect			
AIS	NLR	High vs. low	Mortality	Si-Ying Song, 2019	20/20/0	No serious risk	Serious inconsistency	No serious indirectness	No serious imprecision	NA	Would not reduce effect	No	No	Low
AHS	NLR	High vs. low	Mortality	Si-Ying Song, 2019	20/20/0	No serious risk	Serious inconsistency	No serious indirectness	No serious imprecision	NA	Would not reduce effect	No	No	Low
STEMI undergoing PCI	NLR	High vs. low	In-hospital ACM	Hassan Ul Hussain, 2024	18/18/0	No serious risk	No serious inconsistency	No serious indirectness	No serious imprecision	Strongly suspected	Would not reduce effect	Yes	No	Low
STEMI undergoing PCI	NLR	High vs. low	long-term MACE	Hassan Ul Hussain, 2024	6/6/0	No serious risk	No serious inconsistency	No serious indirectness	No serious imprecision	NA	Would not reduce effect	Yes	No	Moderate
Acute	NLR	High vs.	Poor functional	Jinzhao Wan,	13/13/0	No serious	Serious inconsi	No serious	No serious	Strongly	Would not	No	No	Very

stroke		low	outcome at 3 months	2020		risk	stency	indirectness	imprecision	suspected	reduce effect			low
Acute stroke	NLR	High vs. low	Mortality at 3 months	Jinzhao Wan, 2020	13/13/0	No serious risk	Serious inconsistency	No serious indirectness	Serious imprecision	Strongly suspected	Would not reduce effect	No	No	Very low
AIS treated with IVT	NLR	High vs. low	HT	Chengbing Wang, 2021	6/6/0	No serious risk	Serious inconsistency	No serious indirectness	No serious imprecision	Strongly suspected	Would not reduce effect	No	No	Very low
AIS treated with IVT	NLR	High vs. low	Poor functional outcome at 3 months	Chengbing Wang, 2021	10/10/0	No serious risk	Serious inconsistency	No serious indirectness	Serious imprecision	Strongly suspected	Would not reduce effect	No	No	Very low
AIS treated with RT	Admission NLR	High vs. low	PFO	Bing Wu, 2023	26/26/0	No serious risk	Serious inconsistency	No serious indirectness	No serious imprecision	Strongly suspected	Would not reduce effect	No	No	Very low

AIS treated with RT	Post-treatment NLR	High vs. low	PFO	Bing Wu, 2023	14/14/0	No serious risk	Serious inconsistency	No serious indirectness	No serious imprecision	Strongly suspected	Would not reduce effect	No	No	Very low
AIS treated with RT	Admission NLR	High vs. low	sICH	Bing Wu, 2023	16/16/0	No serious risk	Serious inconsistency	No serious indirectness	No serious imprecision	Strongly suspected	Would not reduce effect	No	No	Very low
AIS treated with RT	Post-treatment NLR	High vs. low	sICH	Bing Wu, 2023	4/4/0	No serious risk	Serious inconsistency	No serious indirectness	Serious imprecision	Strongly suspected	Would not reduce effect	No	No	Very low
AIS treated with RT	Admission NLR	High vs. low	3-month mortality	Bing Wu, 2023	14/14/0	No serious risk	Serious inconsistency	No serious indirectness	No serious imprecision	Strongly suspected	Would not reduce effect	No	No	Very low
AIS treated with RT	Post-treatment NLR	High vs. low	3-month mortality	Bing Wu, 2023	6/6/0	No serious risk	Serious inconsistency	No serious indirec	No serious impreci	NA	Would not reduce	No	No	Low

								tness	sion		effect			
ACS	SII	High vs. low	Short-term ACM	Shengpeng Wang, 2024	11/11/0	No serious risk	Serious inconsistency	No serious indirectness	No serious imprecision	NA	Would not reduce effect	Yes	No	Moderate
ACS	SII	High vs. low	Long-term ACM	Shengpeng Wang, 2024	11/11/0	No serious risk	Serious inconsistency	No serious indirectness	No serious imprecision	NA	Would not reduce effect	Yes	No	Moderate
ACS	SII	High vs. low	Short-term MACEs	Shengpeng Wang, 2024	11/11/0	No serious risk	No serious inconsistency	No serious indirectness	No serious imprecision	NA	Would not reduce effect	No	No	Low
ACS	SII	High vs. low	Long-term MACEs	Shengpeng Wang, 2024	11/11/0	No serious risk	Serious inconsistency	No serious indirectness	No serious imprecision	Strongly suspected	Would not reduce effect	Yes	No	Low
Stroke	SII	High vs.	Poor	Yong-Wei	19/19/0	No serious	Serious inconsi	No serious	Serious impreci	NA	Would not	No	No	Very

		low	outcome	Huang, 2022		risk	stency	indirectness	sion		reduce effect			low
Stroke	SII	High vs. low	Mortality	Yong-Wei Huang, 2022	19/19/0	No serious risk	No serious inconsistency	No serious indirectness	No serious imprecision	NA	Would not reduce effect	Yes	No	Moderate
Stroke	SII	High vs. low	HT	Yong-Wei Huang, 2022	19/19/0	No serious risk	No serious inconsistency	No serious indirectness	No serious imprecision	NA	Would not reduce effect	Yes	No	Moderate
Different levels of SII	SII	Dose-response relationships	ACM	Wei Li, 2024	33/33/0	No serious risk	Serious inconsistency	No serious indirectness	Serious imprecision	Strongly suspected	Would not reduce effect	No	No	Very low
Different levels of SII	SII	Linear dose-response relationship	CVD mortality	Wei Li, 2024	11/11/0	No serious risk	Serious inconsistency	No serious indirectness	Serious imprecision	Undetected	Would not reduce effect	No	No	Very low

Different levels of SII	SII	High vs. low	CVD risk	Zhen Ye, 2022	8/8/0	No serious risk	Serious inconsistency	No serious indirectness	Serious imprecision	Undetected	Would not reduce effect	No	No	Very low
Patients undergoing PCI	SII	High vs. low	MACEs	Chunyu Zhang, 2024	8/8/0	Serious imprecision	No serious inconsistency	No serious indirectness	No serious imprecision	NA	Would not reduce effect	Yes	No	Low
Patients undergoing PCI	SII	High vs. low	ACM	Chunyu Zhang, 2024	3/3/0	Serious imprecision	Serious inconsistency	No serious indirectness	No serious imprecision	NA	Would not reduce effect	Yes	No	Very low
Patients undergoing PCI	SII	High vs. low	Non-fatal MI	Chunyu Zhang, 2024	4/4/0	Serious imprecision	Serious inconsistency	No serious indirectness	No serious imprecision	NA	Would not reduce effect	No	No	Very low
Patients undergoing PCI	SII	High vs. low	HF	Chunyu Zhang, 2024	3/3/0	Serious imprecision	No serious inconsi	No serious indirec	No serious impreci	NA	Would not reduce	No	No	Very low

							stency	tness	sion		effect			
CAD	SII	High vs. low	MACE	Zehao Zhao, 2024	6/6/0	No serious risk	Serious inconsistency	No serious indirectness	No serious imprecision	Strongly suspected	Would not reduce effect	Yes	No	Low
CAD	SII	High vs. low	ACM	Zehao Zhao, 2024	4/4/0	No serious risk	Serious inconsistency	No serious indirectness	No serious imprecision	No serious imprecision	Would not reduce effect	Yes	No	Moderate
CAD	SII	High vs. low	Cardiovascular mortality	Zehao Zhao, 2024	3/3/0	No serious risk	Serious inconsistency	No serious indirectness	No serious imprecision	NA	Would not reduce effect	Yes	No	Moderate
CAD	SII	High vs. low	MI	Zehao Zhao, 2024	4/4/0	No serious risk	Serious inconsistency	No serious indirectness	Serious imprecision	NA	Would not reduce effect	No	No	Very low

CAD	SII	High vs. low	Stroke	Zehao Zhao, 2024	3/3/0	No serious risk	Serious inconsi stency	No serious indirec tness	Serious impreci sion	NA	Would not reduce effect	Yes	No	Low
STEMI patients after pPCI	PLR	High vs. low	MACE (In-hospita l)	Guoxia Dong, 2021	6/6/0	No serious risk	No serious inconsi stency	No serious indirec tness	No serious impreci sion	NA	Would not reduce effect	No	No	Low
STEMI patients after pPCI	PLR	High vs. low	ACM (In-hospita l)	Guoxia Dong, 2021	2/2/0	No serious risk	No serious inconsi stency	No serious indirec tness	No serious impreci sion	NA	Would not reduce effect	No	No	Low
STEMI patients after pPCI	PLR	High vs. low	cardiac mortality (In-hospita l)	Guoxia Dong, 2021	7/7/0	No serious risk	No serious inconsi stency	No serious indirec tness	Serious impreci sion	NA	Would not reduce effect	Yes	No	Low
STEMI patients after pPCI	PLR	High vs. low	no reflow after PCI (In-hospita	Guoxia Dong, 2021	6/6/0	No serious risk	Serious inconsi stency	No serious indirec	No serious impreci	NA	Would not reduce	Yes	No	Moder ate

			l)					tness	sion		effect			
STEMI patients after pPCI	PLR	High vs. low	MACE (Long-term)	Guoxia Dong, 2021	4/4/0	No serious risk	Serious inconsistency	No serious indirectness	No serious imprecision	NA	Would not reduce effect	No	No	Low
STEMI patients after pPCI	PLR	High vs. low	ACM (Long-term)	Guoxia Dong, 2021	5/5/0	No serious risk	Serious inconsistency	No serious indirectness	No serious imprecision	NA	Would not reduce effect	Yes	No	Moderate
AIS treated with RT	Admission PLR	High vs. low	90-day Good Functional Outcomes	Divyansh Sharma, 2022	12/12/0	No serious risk	Serious inconsistency	No serious indirectness	Serious imprecision	NA	Would not reduce effect	No	No	Very low
AIS treated with RT	Delayed PLR	High vs. low	90-day Good Functional Outcomes	Divyansh Sharma, 2022	12/12/0	No serious risk	No serious inconsistency	No serious indirectness	No serious imprecision	NA	Would not reduce effect	No	No	Low
AIS treated	PLR	High vs.	ENI	Divyansh	12/12/0	No serious	No serious	No serious	Serious impreci	NA	Would not	No	No	Very

with RT		low		Sharma, 2022		risk	inconsistency	indirectness	sion		reduce effect			low
HF	PLR	High vs. low	Follow-up mortality	Mehrbod Vakhshoori, 2024	16/16/0	No serious risk	Serious inconsistency	No serious indirectness	Serious imprecision	Strongly suspected	Would not reduce effect	No	No	Very low
HF	PLR	High vs. low	In-hospital mortality	Mehrbod Vakhshoori, 2024	5/5/0	No serious risk	Serious inconsistency	No serious indirectness	No serious imprecision	Strongly suspected	Would not reduce effect	No	No	Very low
AIS	SIRI	High vs. low	Poor functional outcome at 3 months	Ying Han and Nan Lin, 2024	14/14/0	No serious risk	No serious inconsistency	No serious indirectness	No serious imprecision	Undetected	Would not reduce effect	No	No	Low
AIS	SIRI	High vs. low	Poor functional outcome	Yong-Wei Huang, 2022	11/11/0	No serious risk	Serious inconsistency	No serious indirectness	Serious imprecision	NA	Would not reduce effect	Yes	No	Low

AIS	SIRI	High vs. low	SAP	Yong-Wei Huang, 2022	2/2/0	No serious risk	No serious inconsistency	No serious indirectness	No serious imprecision	NA	Would not reduce effect	Yes	No	Moderate
AIS	SIRI	High vs. low	END	Yong-Wei Huang, 2022	2/2/0	No serious risk	Serious inconsistency	No serious indirectness	No serious imprecision	NA	Would not reduce effect	Yes	No	Moderate
Non-significant associations														
Cardiac Surgery	Postoperative NLR	High vs. low	POAF	Zhengyang Liu, 2020	12/12/0	No serious risk	Serious inconsistency	No serious indirectness	No serious imprecision	Undetected	Would not reduce effect	No	No	Low
AIS treated with IVT	NLR	High vs. low	3-month mortality	Chengbing Wang, 2021	4/4/0	No serious risk	Serious inconsistency	No serious indirectness	Serious imprecision	Strongly suspected	Would not reduce effect	No	No	Very low
NSTEMI	NLR	High vs.	Mortality	Michał Pruc,	3/3/0	Serious	Serious inconsi	No serious	Serious impreci	Strongly	Would not	No	No	Very

		low		2024		risk	stency	indirectness	sion	suspected	reduce effect			low
PAD	NLR	High vs. low	4-year MALE	Roy B. Kurniawan, 2024	6/6/0	No serious risk	No serious inconsistency	No serious indirectness	No serious imprecision	NA	Would not reduce effect	No	No	Low
AAD	NLR	High vs. low	NLR values in the hospital death team compared to the survival team	Zekun Li, 2023	8/0/8	No serious risk	No serious inconsistency	No serious indirectness	Serious imprecision	Undetected	Would not reduce effect	No	No	Very low
AAD	NLR	High vs. low	In-hospital mortality	Zekun Li, 2023	8/0/8	No serious risk	Serious inconsistency	No serious indirectness	Serious imprecision	Undetected	Would not reduce effect	No	No	Very low

Atrial Fibrillation Ablation	Preablation NLR	High vs. low	AF recurrence	Sai Prasanna Lekkala, 2023	3/3/0	No serious risk	Serious inconsistency	No serious indirectness	Serious imprecision	Undetected	Would not reduce effect	No	No	Very low
Atrial Fibrillation Ablation	Postablation NLR	High vs. low	AF recurrence	Sai Prasanna Lekkala, 2023	2/2/0	No serious risk	Serious inconsistency	No serious indirectness	Serious imprecision	Undetected	Would not reduce effect	No	No	Very low
AHS	NLR	High vs. low	Functional outcome	Si-Ying Song, 2019	17/17/0	No serious risk	Serious inconsistency	No serious indirectness	No serious imprecision	NA	Would not reduce effect	No	No	Low
STEMI undergoing PCI	NLR	High vs. low	Long-term any revascularization	Hassan Ul Hussain, 2024	4/4/0	No serious risk	No serious inconsistency	No serious indirectness	No serious imprecision	NA	Would not reduce effect	No	No	Low
HF	PLR	High vs. low	Long-term mortality risk	Mehrbod Vakhshoori, 2024	NA	No serious risk	Serious inconsistency	No serious indirec	Serious imprecision	Strongly suspe	Would not reduce	No	No	Very low

								tness		cted	effect			
Patients undergoing cardiothoracic surgery	PLR	High vs. low	POAF incidence after all surgeries	Biling Ye, 2024	6/1/5	No serious risk	No serious inconsistency	No serious indirectness	Serious imprecision	Undetected	Would not reduce effect	No	No	Very low
Patients undergoing cardiothoracic surgery	PLR	High vs. low	POAF incidence specifically after CABG	Biling Ye, 2024	6/1/5	No serious risk	Serious inconsistency	No serious indirectness	Serious imprecision	Undetected	Would not reduce effect	No	No	Very low
Stroke	SII	High vs. low	Recanalization	Yong-Wei Huang, 2022	19/19/0	No serious risk	Serious inconsistency	No serious indirectness	No serious imprecision	NA	Would not reduce effect	No	No	Low
Patients undergoing PCI	SII	High vs. low	Non-fatal stroke	Chunyu Zhang, 2024	3/3/0	No serious risk	Serious inconsistency	No serious indirectness	No serious imprecision	NA	Would not reduce effect	No	No	Low

Patients undergoing PCI	SII	High vs. low	Repeat revascularization	Chunyu Zhang, 2024	4/4/0	No serious risk	Serious inconsistency	No serious indirectness	No serious imprecision	NA	Would not reduce effect	No	No	Very low
CAD	SII	High vs. low	Ischemia-driven revascularization	Zehao Zhao, 2024	3/3/0	No serious risk	Serious inconsistency	No serious indirectness	Serious imprecision	NA	Would not reduce effect	No	No	Very low
AIS treated with RT	PLR	High vs. low	Radiological Bleed	Divyansh Sharma, 2022	12/12/0	No serious risk	Serious inconsistency	No serious indirectness	Serious imprecision	NA	Would not reduce effect	No	No	Very low
AIS	SIRI	High vs. low	ACM	Yong-Wei Huang, 2022	4/4/0	No serious risk	Serious inconsistency	No serious indirectness	No serious imprecision	NA	Would not reduce effect	No	No	Low

Note: CI = confidence interval; OR = odds ratio; HR = hazard ratio; RR = risk ratio; SMD = standardized mean difference; WMD = weighted mean difference; MD = mean difference; MA metric = meta-analysis metric; NA = not available or not applicable; T = total number of studies; C = cohort studies; P = population-based case-control and/or cross-sectional studies; CVD = cardiovascular disease; CAD = coronary artery disease; ACS = acute coronary syndrome; MI = myocardial infarction; STEMI = ST-elevation myocardial infarction; NSTEMI = non-ST-elevation myocardial infarction; PAD = peripheral artery disease; AAD = acute aortic dissection; PCI = percutaneous coronary intervention; pPCI = primary percutaneous coronary intervention; CABG = coronary artery bypass grafting; HF = heart failure; MACE = major adverse cardiovascular events; ACM = all-cause mortality; MALE = major adverse limb events; AIS = acute ischemic stroke; AHS = acute

hemorrhagic stroke; aSAH = aneurysmal subarachnoid hemorrhage; HT = hemorrhagic transformation; sICH = symptomatic intracerebral hemorrhage; END = early neurological deterioration; SAP = stroke-associated pneumonia; PSI = pneumonia severity index; PSP = post-stroke pneumonia; ENI = early neurological improvement; PFO = patent foramen ovale; AF = atrial fibrillation; POAF = postoperative atrial fibrillation; IVT = intravenous thrombolysis; RT = reperfusion therapy; IVIG = intravenous immunoglobulin; DCI = delayed cerebral ischemia; Recanalization = restoration of blood flow; NLR = neutrophil-to-lymphocyte ratio; PLR = platelet-to-lymphocyte ratio; SII = systemic immune-inflammation index; SIRI = systemic inflammatory response index; vs.=versus; GRADE=Grading of Recommendations Assessment, Development, and Evaluation; *Category = Immune-inflammatory indices.

6. Supplementary References

Note: The reference list in this Supplementary Material was generated using EndNote 21 (Clarivate Analytics). Minor variations may exist compared with the proofed main manuscript. The final, proof-edited reference list in the main text should be considered authoritative.

1. Angkananard T, Anothaisintawee T, McEvoy M, Attia J, Thakkinstian A. Neutrophil Lymphocyte Ratio and Cardiovascular Disease Risk: A Systematic Review and Meta-Analysis. *Biomed Res Int* 2018;**2018**:2703518. doi: https://doi.org/10.1155/2018/2703518
2. Banahene NO, Sinha T, Shaikh S, Zin AK, Khreis K, Chaudhari SS, et al. Effect of Elevated Neutrophil-to-Lymphocyte Ratio on Adverse Outcomes in Patients With Myocardial Infarction: A Systematic Review and Meta-Analysis. *Cureus* 2024;**16**:e61647. doi: https://doi.org/10.7759/cureus.61647
3. Jackson SM, Perry LA, Borg C, Ramson DM, Campbell R, Liu Z, et al. Prognostic Significance of Preoperative Neutrophil-Lymphocyte Ratio in Vascular Surgery: Systematic Review and Meta-Analysis. *Vasc Endovascular Surg* 2020;**54**:697-706. doi: https://doi.org/10.1177/1538574420951315
4. Khanzadeh S, Lucke-Wold B, Eshghyar F, Rezaei K, Clark A. The Neutrophil to Lymphocyte Ratio in Poststroke Infection: A Systematic Review and Meta-Analysis. *Dis Markers* 2022;**2022**:1983455. doi: https://doi.org/10.1155/2022/1983455
5. Kurniawan RB, Siahaan PP, Saputra PB, Arnindita JN, Savitri CG, Faizah NN, et al. Neutrophil-to-lymphocyte ratio as a prognostic biomarker in patients with peripheral artery disease: A systematic review and meta-analysis. *Vasc Med* 2024;**29**:687-699. doi: https://doi.org/10.1177/1358863X241281699
6. Liu Z, Nguyen Khuong J, Borg Caruana C, Jackson SM, Campbell R, Ramson DM, et al. The Prognostic Value of Elevated Perioperative Neutrophil-Lymphocyte Ratio in Predicting Postoperative Atrial Fibrillation After Cardiac Surgery: A Systematic Review and Meta-Analysis. *Heart Lung Circ* 2020;**29**:1015-1024. doi: https://doi.org/10.1016/j.hlc.2019.11.021
7. Shahsanaei F, Abbaszadeh S, Behrooj S, Rahimi Petrudi N, Ramezani B. The value of neutrophil-to-lymphocyte ratio in predicting severity of coronary involvement and long-term outcome of percutaneous coronary intervention in patients with acute coronary syndrome: a systematic review and meta-analysis. *Egypt Heart J* 2024;**76**:39. doi: https://doi.org/10.1186/s43044-024-00469-3

8. Shi M, Yang C, Tang QW, Xiao LF, Chen ZH, Zhao WY. The Prognostic Value of Neutrophil-to-Lymphocyte Ratio in Patients With Aneurysmal Subarachnoid Hemorrhage: A Systematic Review and Meta-Analysis of Observational Studies. *Front Neurol* 2021;**12**:745560. doi: https://doi.org/10.3389/fneur.2021.745560
9. Wang X, Zhang G, Jiang X, Zhu H, Lu Z, Xu L. Neutrophil to lymphocyte ratio in relation to risk of all-cause mortality and cardiovascular events among patients undergoing angiography or cardiac revascularization: a meta-analysis of observational studies. *Atherosclerosis* 2014;**234**:206-213. doi: https://doi.org/10.1016/j.atherosclerosis.2014.03.003
10. Xu Y, Fang H, Qiu Z, Cheng X. Prognostic role of neutrophil-to-lymphocyte ratio in aortic disease: a meta-analysis of observational studies. *J Cardiothorac Surg* 2020;**15**:215. doi: https://doi.org/10.1186/s13019-020-01263-3
11. Zhang R, Wu X, Hu W, Zhao L, Zhao S, Zhang J, et al. Neutrophil-to-lymphocyte ratio predicts hemorrhagic transformation in ischemic stroke: A meta-analysis. *Brain Behav* 2019;**9**:e01382. doi: https://doi.org/10.1002/brb3.1382
12. Dentali F, Nigro O, Squizzato A, Gianni M, Zuretti F, Grandi AM, Guasti L. Impact of neutrophils to lymphocytes ratio on major clinical outcomes in patients with acute coronary syndromes: A systematic review and meta-analysis of the literature. *Int J Cardiol* 2018;**266**:31-37. doi: https://doi.org/10.1016/j.ijcard.2018.02.116
13. Li W, Hou M, Ding Z, Liu X, Shao Y, Li X. Prognostic Value of Neutrophil-to-Lymphocyte Ratio in Stroke: A Systematic Review and Meta-Analysis. *Front Neurol* 2021;**12**:686983. doi: https://doi.org/10.3389/fneur.2021.686983
14. Lu M, Zhang Y, Liu R, He X, Hou B. Predictive value of neutrophil to lymphocyte ratio for ischemic stroke in patients with atrial fibrillation: A meta-analysis. *Front Neurol* 2022;**13**:1029010. doi: https://doi.org/10.3389/fneur.2022.1029010
15. Mikhael R, Hindoro E, Taner S, Lukito AA. Neutrophil-to-lymphocyte ratio for predictor of in-hospital mortality in ST-segment elevation myocardial infarction: a meta-analysis. *Medical Journal of Indonesia* 2020;**29**:172-182. doi: https://doi.org/10.13181/mji.oa.202795
16. Pruc M, Kubica J, Banach M, Swieczkowski D, Rafique Z, Peacock WF, et al. Diagnostic and prognostic performance of the neutrophil-to-lymphocyte ratio in acute coronary syndromes: A meta-analysis of 90 studies including 45 990 patients. *Kardiol Pol* 2024;**82**:276-284. doi: https://doi.org/10.33963/v.phj.99554
17. Song SY, Zhao XX, Rajah G, Hua C, Kang RJ, Han YP, et al. Clinical Significance of Baseline Neutrophil-to-Lymphocyte Ratio in Patients With Ischemic Stroke or Hemorrhagic Stroke: An Updated Meta-Analysis. *Front Neurol* 2019;**10**:1032. doi: https://doi.org/10.3389/fneur.2019.01032

18. Ul Hussain H, Kumar KA, Zahid M, Husban Burney M, Khan Z, Asif M, et al. Neutrophil to lymphocyte ratio as a prognostic marker for cardiovascular outcomes in patients with ST-segment elevation myocardial infarction after percutaneous coronary intervention: A systematic review and meta-analysis. *Medicine (Baltimore)* 2024;**103**:e38692. doi: https://doi.org/10.1097/MD.0000000000038692
19. Wan J, Wang X, Zhen Y, Chen X, Yao P, Liu W, et al. The predictive role of the neutrophil-lymphocyte ratio in the prognosis of adult patients with stroke. *Chin Neurosurg J* 2020;**6**:22. doi: https://doi.org/10.1186/s41016-020-00201-5
20. Wang C, Zhang Q, Ji M, Mang J, Xu Z. Prognostic value of the neutrophil-to-lymphocyte ratio in acute ischemic stroke patients treated with intravenous thrombolysis: a systematic review and meta-analysis. *BMC Neurol* 2021;**21**:191. doi: https://doi.org/10.1186/s12883-021-02222-8
21. Wu B, Liu F, Sun G, Wang S. Prognostic role of dynamic neutrophil-to-lymphocyte ratio in acute ischemic stroke after reperfusion therapy: A meta-analysis. *Front Neurol* 2023;**14**:1118563. doi: https://doi.org/10.3389/fneur.2023.1118563
22. Wang S, Zhang G. Association Between Systemic Immune-Inflammation Index and Adverse Outcomes in Patients With Acute Coronary Syndrome: A Meta-Analysis. *Angiology* 2024:33197241263399. doi: https://doi.org/10.1177/00033197241263399
23. Huang YW, Yin XS, Li ZP. Association of the systemic immune-inflammation index (SII) and clinical outcomes in patients with stroke: A systematic review and meta-analysis. *Front Immunol* 2022;**13**:1090305. doi: https://doi.org/10.3389/fimmu.2022.1090305
24. Li W, Wang X, Diao H, Yang Y, Ding L, Huan W, et al. Systemic immune inflammation index with all-cause and cause-specific mortality: a meta-analysis. *Inflamm Res* 2024;**73**:2199-2216. doi: https://doi.org/10.1007/s00011-024-01959-5
25. Ye Z, Hu T, Wang J, Xiao R, Liao X, Liu M, Sun Z. Systemic immune-inflammation index as a potential biomarker of cardiovascular diseases: A systematic review and meta-analysis. *Front Cardiovasc Med* 2022;**9**:933913. doi: https://doi.org/10.3389/fcvm.2022.933913
26. Zhang C, Li M, Liu L, Deng L, Yulei X, Zhong Y, et al. Systemic immune-inflammation index as a novel predictor of major adverse cardiovascular events in patients undergoing percutaneous coronary intervention: a meta-analysis of cohort studies. *BMC Cardiovasc Disord* 2024;**24**:189. doi: https://doi.org/10.1186/s12872-024-03849-4
27. Zhao Z, Zhang X, Sun T, Huang X, Ma M, Yang S, Zhou Y. Prognostic value of systemic immune-inflammation index in CAD patients: Systematic review and meta-analyses. *Eur J Clin Invest* 2024;**54**:e14100. doi: https://doi.org/10.1111/eci.14100
28. Dong G, Huang A, Liu L. Platelet-to-lymphocyte ratio and prognosis in STEMI: A meta-analysis. *Eur J Clin Invest* 2021;**51**:e13386. doi: https://doi.org/10.1111/eci.13386

29. Sharma D, Bhaskar SMM. Prognostic Role of the Platelet-Lymphocyte Ratio in Acute Ischemic Stroke Patients Undergoing Reperfusion Therapy: A Meta-Analysis. *J Cent Nerv Syst Dis* 2022;**14**:11795735221110373. doi: https://doi.org/10.1177/11795735221110373
30. Vakhshoori M, Bondariyan N, Sabouhi S, Kiani K, Alaei Faradonbeh N, Emami SA, et al. The impact of platelet-to-lymphocyte ratio on clinical outcomes in heart failure: a systematic review and meta-analysis. *Ther Adv Cardiovasc Dis* 2024;**18**:17539447241227287. doi: https://doi.org/10.1177/17539447241227287
31. Han Y, Lin N. Systemic Inflammatory Response Index and the Short-Term Functional Outcome of Patients with Acute Ischemic Stroke: A Meta-analysis. *Neurol Ther* 2024;**13**:1431-1451. doi: https://doi.org/10.1007/s40120-024-00645-2
32. Huang YW, Zhang Y, Feng C, An YH, Li ZP, Yin XS. Systemic inflammation response index as a clinical outcome evaluating tool and prognostic indicator for hospitalized stroke patients: a systematic review and meta-analysis. *Eur J Med Res* 2023;**28**:474. doi: https://doi.org/10.1186/s40001-023-01446-3
33. Li Z, Lian D, Wang R, Li J, Wang W. Meta-analysis of the relationship between neutrophil to lymphocyte ratio and short-term prognosis of patients with acute aortic dissection. *Archives of Clinical Psychiatry* 2023;**50**:143. doi: https://doi.org/10.15761/0101-60830000000604
34. Lekkala SP, Mellacheruvu SP, Gill KS, Khela PS, Singh G, Jitta SR, et al. Association between preablation and postablation neutrophil-lymphocyte ratio and atrial fibrillation recurrence: A meta-analysis. *J Arrhythm* 2024;**40**:214-221. doi: https://doi.org/10.1002/joa3.12996
35. Ye B, Gan J, Han Y, Yu L, Huang Y, Ye B. Relationship between Prediction of Platelet-Lymphocyte Ratio and Atrial Fibrillation in Perioperative Patients: A Systematic Review and Meta-Analysis. *The Heart Surgery Forum* 2024;**27**:E180-E187. doi: https://doi.org/10.59958/hsf.6851
